# Synthesis, characterization, DNA binding interactions, DFT calculations, and Covid-19 molecular docking of novel bioactive copper(I) complexes developed via unexpected reduction of azo-hydrazo ligands

**DOI:** 10.1186/s13065-023-01086-y

**Published:** 2023-11-20

**Authors:** Eman Hassan Elsayed, Dhuha Al-Wahaib, Ali El-Dissouky Ali, Beshir A. Abd-El-Nabey, Hemmat A. Elbadawy

**Affiliations:** 1 Chemistry Department, Faculty of Science, Alexandria University, Alexandria, Egypt; 2 Chemistry Department, Faculty of Science, Kuwait University, Safat, Kuwait

**Keywords:** Redox-active ligands, Copper(i) complexes, XPS, Cyclic voltammetry, Anticancer, DNA binding, SARS-CoV-2

## Abstract

In this work, we focused on the 3rd goal of the sustainable development plan: achieving good health and supporting well-being. Two redox**-**active hydrazo ligands namely, phenylcarbonohydrazonoyldicyanide (PCHD) and pyridin-4-ylcarbonohydrazonoyl-dicyanide (PyCHD), and their copper(I) complexes have been synthesized and characterized. The analytical data indicates the formation of copper(I) complexes despite starting with copper(II) perchlorate salt. The ^1^H-NMR and UV–visible spectral studies in DMSO revealed that PyCHD mainly exists in its azo-form, while PCHD exists in azo ↔ hydrazo equilibrium form, and confirmed the copper(I) oxidation state. XPS, spectral and electrochemistry data indicated the existence of copper(I) valence of both complexes. Cyclic voltammetry of PCHD and its copper(I) complex supported the reduction power of the ligand. The antimicrobial activity, cytotoxicity against the mammalian breast carcinoma cell line (MCF7), and DNA interaction of the compounds are investigated. All compounds showed high antimicrobial, and cytotoxic activities, relative to the standard drugs. Upon studying the wheat DNA binding, PCHD and PyCHD were found to bind through external contacts, while both [Cu(PCHD)_2_]ClO_4_.H_2_O and [Cu(PyCHD)_2_]ClO_4_.H_2_O were intercalated binding. In-silico molecular docking simulations against Estrogen Receptor Alpha Ligand Binding Domain (ID: 6CBZ) were performed on all produced compounds and confirmed the *invitro* experimentally best anticancer activity of [Cu(PyCHD)_2_]ClO_4_.H_2_O. The molecular docking tests against SARS-CoV-2 main protease (ID: 6 WTT) showed promising activity in the order of total binding energy values: [Cu(PCHD)_2_]ClO_4_.H_2_O > [Cu(PyCHD)_2_]ClO_4_.H_2_O > PCHD > PyCHD.

**Supplementary Information:**

The online version contains supplementary material available at 10.1186/s13065-023-01086-y.

## Introduction

The spread of cancerous diseases is a major contributor to the global health crisis. These issues have recently been compounded by the Corona pandemic, which is devastatingly affecting humanity. Consequently, an argent request was made to design and synthesize drugs, then investigate their in vitro and in vivo biological effects. Recently, computational, and molecular docking studies have been used frequently to design medications and conduct hypothetical tests to predict their efficacy [1–3].

Hydrazo-containing compounds represent a unique and important class of Schiff bases with the functional group -C = N–NH-. These compounds are of particular significance due to their notable redox properties, stability, wide range of biological activities, and commercial utility. Additionally, their affordability and straightforward synthesis further contribute to their appeal [4, 5]. The interaction of hydrazo compounds with metal ions, produces metal complexes possessing unique features, particularly in the field of antibacterial and antifungal activities [6–9]. Stable metal complexes fragment with high delocalization of the electron density are prevalent amongst coordination compounds. The remarkable redox activity exhibited by azo-hydrazones has played a crucial role in the formation of metal complexes, leading to potent medicinal and biological consequences [8].

In chemistry and materials-related researches, density-functional theory (DFT) became a common computational method used to investigate the electronic and nuclear structure of molecules in their ground state. DFT considers functionals that are related to the spatially varying electron density, giving a view about multi-electron systems. However, several disciplines, including computational chemistry, accept DFT as an important method due to its versatility and widespread application [10–12]. Combination between computational studies and experimental data give better understanding of the material properties, which may lead to different applications. Moreover, Molecular docking (MD) is known to be a low-cost theoretical modeling method employed to simulate the action between designed drugs and cell receptors. This is a very helpful method that can be applied before experimental trials to select the most expected useful drug. Recently, MD was employed to examine several drugs against corona virus helping in the development of potential medicines or drugs for the treatment of infection or symptoms [13, 14].

In this research, and in continuation of our work for the study of azo-hydrazo ligands` coordination behavior [8, 9, 15, 16], we are studying the behavior of aniline and 4-aminopyridine based azo-hydrazo compounds toward copper(II) perchlorate salt, characterizing the products via different physicochemical tools, studying their bioactivity, and DNA binding mode. Furthermore, the DFT computational studies are applied to simulate and describe the molecular structure and some biological properties based on the electronic properties of the compounds. In addition, the utilization of molecular docking is employed in two distinct directions. Firstly, it is utilized to gain insights into the molecular interaction and binding efficacy of the products towards the in vitro examined breast cancer. This is achieved by employing the Estrogen Receptor Alpha Ligand Binding Domain (PDB Id: 6CBZ) as a reference. Secondly, molecular docking is employed to investigate the potential of synthesized compounds as a drug for SARS-CoV-2, a member of the coronavirus family. SARS-CoV-2 is composed of four structural proteins and sixteen non-structural proteins (NSP). Among these, the main protease (M^pro^), papain-like protease (PLpro), and RNA dependent RNA polymerase (RdRp) are believed to play a crucial role in the replication of the virus [17–19]. Thus, the molecular docking targeting M^pro^ is utilized to predict their efficiency to inhibit its growth.

## Experimental

Materials and reagents as well as physical measurements; CHN and metal analyses, molar conductivity, spectral analysis (FT-IR, UV–Vis,^1^H-NMR, as well as X-ray photoemission spectroscopy), and cyclic voltammetry are described in supplementary materials. All chemicals were used referring to their MSDS sheets. Many trials were performed to obtain crystals using different solvents, but unfortunately stable crystals suitable for single crystal x-ray analysis were not isolated. However, a number of techniques were employed to fully describe and illustrate the compounds.

### Synthesis of ligands and their copper complexes

#### Synthesis of organic ligands, PCHD and PyCHD

The two ligands, phenylcarbonohydrazonoyl dicyanide (PCHD) and pyridin-4-ylcarbonohydrazonoyl dicyanide (PyCHD) were synthesized similar to literature [8, 9]. Synthesis of PCHD (*C*_*9*_*H*_*6*_*N*_*4*_*)*was described in our previous work as one of the steps leading to the targeted ligand [8]. The same procedure was followed for the synthesis of PyCHD by dissolving 30.0 mmol of 4-aminopyridine (2.82 *g*), in 10 mL of 34% hydrochloric acid. The reaction temperature was kept in the range (-5—0) ℃, while drop wise addition of 20.0 mL aqueous, cold solution NaNO_2_ (2.07 *g*, 30 mmol) with continuous stirring. The product was then slowly added to (10 mL) of malononitrile (1.98 *g*, 30 mmol) and CH_3_COONa (5 *g*, 60 mmol). Next, the isolation of the formed yellow solid was followed by decantation, washing with distilled water, and drying under vacuum at 60 ℃ for 24 h.

#### Synthesis of Bis(N-phenylcarbonohydrazonoyldicyanide)copper(I) perchlorate monohydrate and Bis(pyridin-4-ylcarbonohydrazonoyl-dicyanide) copper(I) perchlorate monohydrate complexes: [Cu(L)_2_]ClO_4_.H_2_O, L = PCHD or PyCHD

A solution of Cu(ClO_4_)_2_.6H_2_O (0.37 mg, 1.0 mmol) in ethanol (10 mL) was slowly dropwise added to a hot ethanolic solution of 15 mL of PCHD (0.34 *g*, 2.0 mmol) or PyCHD (0.35 mg, 2.0 mmol). Stirring the mixture for three hours under reflux at 70 ℃, leaving the products to cool at room temperature, the produced powder filtered, washed with distilled water, and dried in a vacuum oven at 60 ℃ for 24 h.

### Computational calculations

DFT calculations was performed using the hybrid correlation function (B3LYP), with the fitted basis set 6–311G +  + (d.p) with LANL2DZ via Gaussian 09 software package [20, 21]. The Gauss View 6.0 software and Chemcraft program were utilized for drawing the optimized structure of the synthesized ligands and their copper complexes [22]. The optimized geometry energy minima of the products were identified as there were no imaginary frequency modes [23, 24].

### In vitro antimicrobial screening

The antibacterial and antifungal activities of the synthesized compounds: PCHD, PyCHD, [Cu(PCHD)_2_]ClO_4_.H_2_O and [Cu(PyCHD)_2_]ClO_4_.H_2_O were investigated by applying a modified Kirby-Bauer disc diffusion method[25], against some pathogenic bacterial strain. The methodology and process of evaluation are included in the Additional file 1 [26]. [details are described in Additional file 1].

### Cytotoxicity evaluation of the compounds

The cytotoxicity of the four synthesized compounds: PCHD, PyCHD, [Cu(PCHD)_2_]ClO_4_.H_2_O and [Cu(PyCHD)_2_]ClO_4_.H_2_O, was studied against the mammalian breast carcinoma cell line (MCF7). Cell toxicity was determined by assessing the impact of the investigated compounds on cell shape and viability. For cytotoxicity assay, the cells were incubated in a 96-well plate with a cell concentration per well is 1.0 × 10^4^ in 100 μL of growth medium. The cell cytotoxic effects were calculated for each compound [27]. The IC_50_, inhibitory activity, for Cisplatin Standard against MCF-7 was also detected. The method was performed as previously reported [28]. [See Additional file 1].

### DNA-binding studies by UV-absorption spectroscopy

A stock solution of wheat DNA was made by dilution with buffer solution consisting of 150 mM to 15 mM of the NaCl to trisodium citrate, at pH 7.0. The stock solution of wheat DNA was appropriately free of protein contamination that is proved by the value of UV- absorption ratio (260 nm/280 nm) = 1.73 [29–31]. Details are described in the Additional file 1.

### Molecular docking

Molecular docking studies were done to understand the molecular affinity between the synthesized ligands and their copper complexes, against Estrogen Receptor Alpha Ligand Binding Domain (PDB Id: 6CBZ) and COVID-19 M^pro^ of corona virus (PDB Id: 6WTT) using AutoDock4.2 [32]. The three-dimensional crystallographic structure of protein was obtained from PDB website. The antiviral activity of the reference inhibitor GC-376 (K36) was used as an effective docking analysis standard and EST used as a standard for Estrogen Receptor Alpha Ligand Binding Domain. The structure of the receptors was assumed by addition of polar hydrogens and removing the small ligands and ions. Kollman charges were used to calculate the partial atomic charge. The active sites were selected for grid box preparation and adapted with the spacing of 0.592 and 0.375 Å, dimensions of x, y, and z-axes were set to 78 × 96 × 64 and 38 × 32 × 38 points for 6WTT and 6CBZ, respectively. The Lamarckian Genetic Algorithm (LGA) was chosen [33]. The genetic algorithm parameters were set to 50 runs with 300 population size; the number of evaluations was selected to be 25,000,00. Biovia Discovery Studio and Chimera X 1.4 softwares [34] used to visualize the different molecular interactions between the biologically active compounds and the receptor.

## Results and discussion

### Synthesis and characterization of PCHD, PyCHD and their copper(I) complexes

**Scheme 1:**
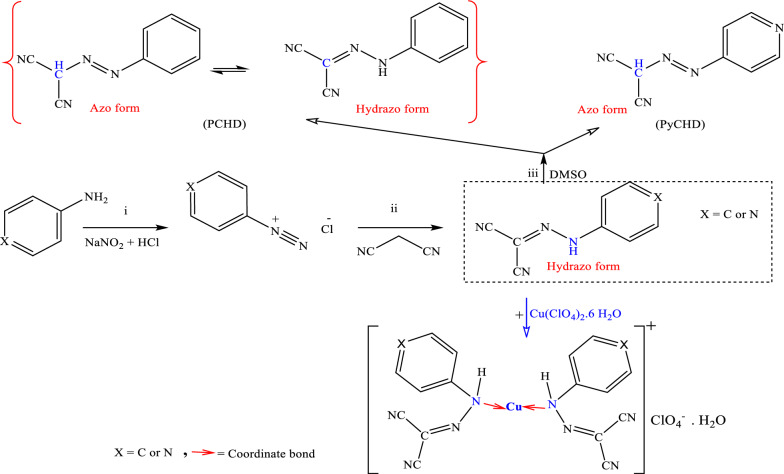
Synthesis of PCHD, PyCHD, [Cu(PCHD)_2_]ClO_4_.H_2_O and [Cu(PyCHD)_2_]ClO_4_.H_2_O

PCHD and PyCHD were synthesized by diazotization of aniline and 4-aminopyridine, respectively, and coupling with malononitrile, Scheme 1. Both ligands are air-stable for a long time, with melting points at 132.0 ± 1.0 and 200.0 ± 1.0 ℃, for PCHD and PyCHD, respectively. The reaction of both ligands with Cu(ClO_4_)_2_.6H_2_O in a 2 ligand:1Cu(II)- mole ratio using ethanol as a solvent gave the corresponding olive green binary copper complex; [Cu(L)_2_]ClO_4_.H_2_O, where (L = PCHD or PyCHD), Scheme 1. The molecular formulae of the copper(I) complexes depending on their elemental analyses, mass spectral data and molar conductivity values, Table 1. For 1.0 × 10^–3^ M in DMSO solutions at 25 ± 1 °C, the molar conductivity values of 28.27 and 37.69 for [Cu(PCHD)_2_]ClO_4_.H_2_O and [Cu(PyCHD)_2_]ClO_4_.H_2_O, respectively, are characteristic of 1:1 electrolytes, supporting the scheduled chemical formulae of the complexes [15, 35]. The complexes are room temperature- air stable and soluble in polar aprotic solvents (DMF and DMSO), slightly soluble in ethanol and methanol, but insoluble in acetone, diethyl ether, and water.

**Table 1:** Elemental analysis and some physical properties of PCHD, PyCHD, [Cu(PCHD)_2_]ClO_4_. H_2_O and [Cu(PyCHD)_2_]ClO_4_. H_2_O

Compounds	Molecular formula	Molecular weight *g*/mol (m/z) ^a^	Color (% yield)	Molar conductance^b^ (Ω^−1 ^cm^2^mol^−1^)	Elemental analysis (%) calculated (found)			
					C	H	N	Cu
**PCHD**	(C_9_H_6_N_4_)	170.06 (170.08)	Yellow (97%)	–	63.52 (63.50)	3.55 (3.52)	32.92 (32.87)	–
**[Cu(PCHD)**_**2**_**]ClO**_**4**_**.H**_**2**_**O**	(C_18_H_14_N_8_O_5_ClC u)	521.34 (521.03)	Brown (59.5%)	28.27	42.30 (42.36)	3.13 (3.09)	21.93 (21.25)	12.44 (12.29)
**PyCHD**	(C_8_H_5_N_5_)	171.05 (171.34)	Yellow (54%)	–	56.14 (56.42)	2.94 (3.17)	40.92 (41.09)	–
**[Cu(PyCHD)**_**2**_**]ClO**_**4**_**.H**_**2**_**O**	(C_16_H_12_N_10_O_5_ClCu)	523.00 (523.10)	Brown (47.8%)	37.69	37.46 (37.33)	2.73 (2.69)	27.31 (27.28)	12.39 (12.37)

^a^Based on the mass spectra, ^b^ at room temperature

#### FT-IR spectroscopy

FT-IR spectra of the PCHD, PyCHD and their copper(I) complexes are noted in the range of 4000- 400 cm^−1^, Additional file 1: Fig. S1, and the most important bands with their exploratory assignments [36] are summarized in Table 2. The ν (C = N) moiety peak appeared as strong band at 1605, 1634, 1640 and 1607 cm^−1^, in PCHD, PyCHD, [Cu(PCHD)_2_]ClO_4_.H_2_O and [Cu(PyCHD)_2_]ClO_4_.H_2_O, respectively [10]. The amide moiety peak appeared as medium broad band at 3469 and 3440 cm^−1^, for PCHD and PyCHD, respectively, is either red-shifted or blue-shifted in the corresponding complex, supporting their coordination to the copper ion. Their bonding to copper(I) ion is also confirmed by the presence of the new weak-medium band at 482 and 431 cm^−1^ assignable to ν(Cu–N) for [Cu(PCHD)_2_]ClO_4_.H_2_O and [Cu(PyCHD)_2_]ClO_4_.H_2_O, respectively [37]. The spectrum of PyCHD exhibits a medium band at 1563 cm^−1^ (absent in PCHD spectrum) assigned to ν (C = N)_pyridine_ [38]. This band is very slightly shifted to appear at 1560 cm^−1^ in the complex, suggesting its non-bonding to the copper ion. The band due to ν(C≡N) is located at 2232 and 2215 cm^−1^ for PCHD and PyCHD, respectively. Upon complex formation, this band is blue shifted in both complexes, which may be attributable to the effect of the complexed hydrazo group on the force strength of the C≡N. The bands in the ranges of 1200–1640 cm^−1^ are assigned to *δ* (C − N) and ν (C = C) and ν (C − C) [38, 39]. While the bands at 3060 cm^−1^ and 3079 cm^−1^ are assigned to the aromatic ν (C–H) in PCHD and PyCHD, respectively, these bands are very slightly affected by complex formation. The spectrum of [Cu(PCHD)_2_]ClO_4_.H_2_O, displayed one strong band at 1169 and one weak band at 928 cm^−1^, while [Cu(PyCHD)_2_]ClO_4_.H_2_O exhibited a very strong band at 1098(vs) and 929 (w) cm^−1^ characteristic of ionic non-coordinated perchlorate moiety [8, 40]. The existence of the perchlorate group in the ionization sphere was confirmed from the room temperature molar conductivity value of 37.69 Ohm^−1^ cm^2^ mol^−1^ for 1.0 × 10^−3^ M solution.

**Table 2:** FT-IR data (ν, cm^−1^) of PCHD, [Cu(PCHD)_2_]ClO_4_.H_2_O, PyCHD and [Cu(PyCHD)_2_]ClO_4_.H_2_O

Compound	v NH	v (CH)_aromatic_	v (C $\equiv$ N)	v (C = N)	v (C = N)_pyridine_	v (C = C)	v (C-N)	v (Cu–N)	v (ClO_4_)
**PCHD**	3469	3060	2232	1605	–-	1474	1282	–	–
**[Cu(PCHD)**_**2**_**]ClO**_**4**_**.H**_**2**_**O**	3408	3044	2183	1640	–-	1450	1240	482	1169vs 928vs
**PyCHD**	3440	3079	2215	1634	1563	1491	1287	–	–
**[Cu(PyCHD)**_**2**_**]ClO**_**4**_**.H**_**2**_**O**	3476	3060	2226	1607	1560	1435	1278	431	1098vs 929vs

*vs* very strong, *s* strong, *m* medium, *w* weak and *b* broad

#### Nuclear magnetic resonance spectra

**Fig. 1:**
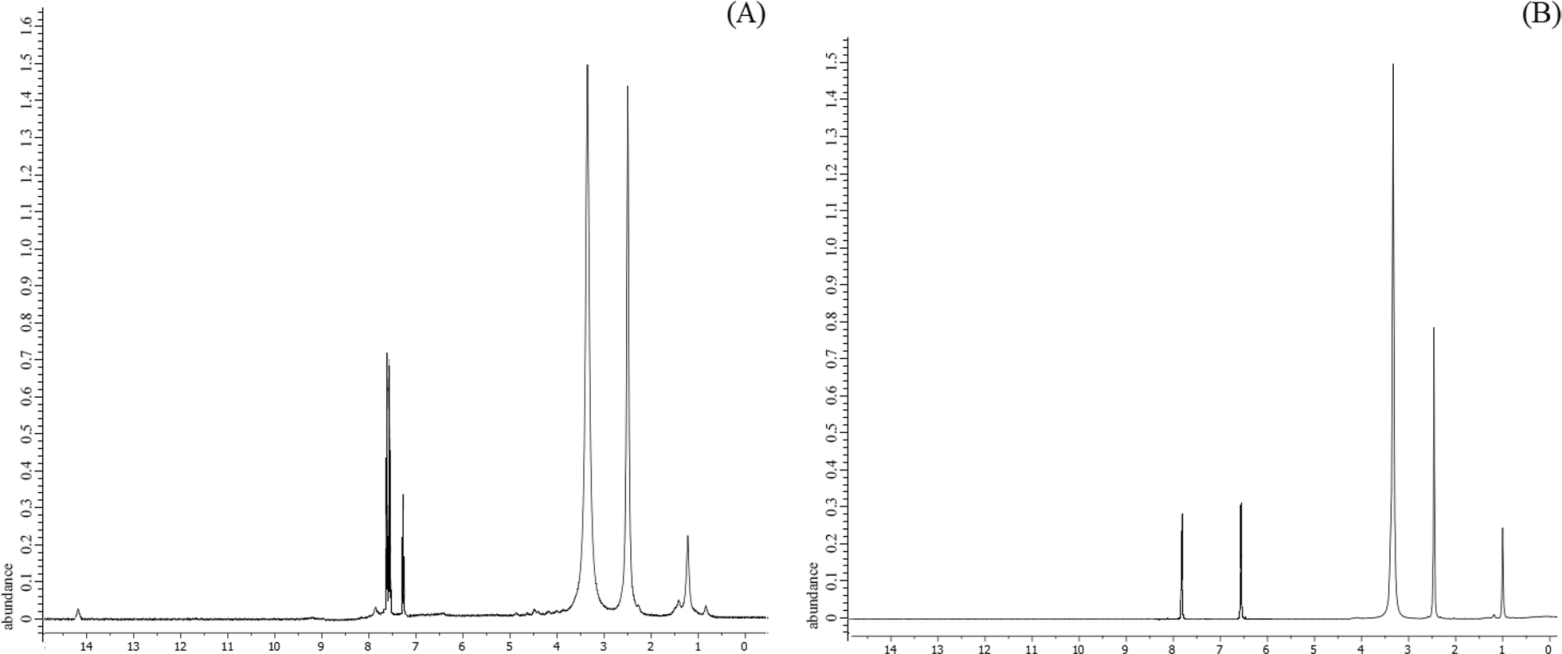
^1^HNMR spectrum of (**A**) [Cu(PCHD)_2_]ClO4.H_2_O and (**B**) [Cu(PyCHD)_2_]ClO_4_.H_2_O complexes in d_6_-DMSO

The ^1^HNMR spectrum of PCHD displays a singlet signal at δ 13.07 ppm (absent in PyCHD), which disappeared in the presence of D_2_O, characteristic of hydrogen-bonded –NH–, Additional file 1: Fig. S2. The two triplet signals at δ 7.12 (one proton) and 7.34 (two protons) ppm belong to para– and meta-protons of the phenyl ring, respectively. The doublet signal at δ 7.39 (two protons) ppm referred to ortho–protons of the phenyl ring. The appearance of (NH) and (CH) signals in ^1^HNMR spectrum of PCHD indicates the co-existence of the azo and hydrazo tautomeric forms, Scheme 1 [41]. Moreover, the suggested tautomerism in PCHD is evidenced by the observation of specific signals in the ^13^C-NMR spectra, Additional file 1: Fig. S3. Specifically, a signal at δC = 84.25 ppm is attributed to the (CH) carbon, while a signal at 142.79 ppm belongs to the (C–NH) carbon. The two cyano-carbons exhibited two distinct signals with chemical shifts of 111.01 and 115.51 ppm. The ^1^HNMR spectrum of PyCHD gave two doublets at δ 7.35 (two protons) and 8.32 (two protons) ppm due to two ortho and two meta protons of the pyridine ring, respectively. The signal at δ 1.89 (one H) ppm in both ligands is attributed to the proton of (CH) moiety. The absence of a peak characteristic of NH proton either at the high field region (δ 6–8 ppm) or low field region (δ > 10 ppm) in the spectrum of PyCHD indicated its existence mainly in the azo-form in DMSO solution. This is confirmed by ^13^C-NMR spectral analysis of PyCHD, Additional file 1: Fig. S3, where it can be deduced that the predominant form of the product is observed to be in the azo configuration. The presence of an imine carbon signal at δC = 77.44 ppm, two cyano-carbons at δC = 116.18 ppm, and pyridyl carbons within the range of (145.66—159.47) ppm are observed. On the other hand, the ^1^HNMR spectra of [Cu(PCHD)_2_]ClO_4_.H_2_O and [Cu(PyCHD)_2_]ClO_4_.H_2_O, Fig. 1, exhibit sharp resonances in the diamagnetic region of the spectrum, which supports the oxidation state assignment of the copper ion as Cu(I).

#### Electronic absorption spectra

PCHD, PyCHD, [Cu(PCHD)_2_]ClO_4_.H_2_O and [Cu(PyCHD)_2_]ClO_4_.H_2_O are subjected to UV–Vis radiation to study their electronic absorption spectra, Additional file 1: Fig. S4, and the data are assembled in Table 3. The spectra of PCHD and PyCHD display intra-ligand π-π* transitions bands at 256 (ε = 10,680 Lmol^−1^ cm^−1^) and 260 nm (ε = 32,480 Lmol^−1^ cm^−1^), respectively. Moreover, the n- π* transitions appear as two bands at 360 and 380 nm with ε = 15,680 and 15,310 Lmol^−1^ cm^−1^, respectively, for PCHD. The spectrum of PyCHD displays only one broad band at 425 nm with ε = 20,468 Lmol^−1^ cm^−1^. The bands at 380 nm for PCHD and at 425 nm for PyCHD are attributed to n- π* transitions involving the entire electronic system with charge-transfer (CT) criteria, which attributed to intramolecular CT transition from the nitrogen atom to the heterocycle moiety. The appearance of a split broad band with maxima at 360 and 380 nm in the spectrum of PCHD indicates the presence of azo-hydrazo tautomeric forms in agreement with ^1^H-NMR outcomes [42]. The d-d transition bands were not observed due to their hidden under the CT band and/or Cu(II) reduction into Cu(I) upon complex formation. The spectra were repeated within two days, showing stability of investigated compounds in DMSO.

**Table 3:** Electronic spectral transitions for PCHD, PyCHD, and their copper complexes

Compound	π–π* λ_max_ (nm) (ε,Lmol^−1^cm^−1^)	n–π* λ_max_ (nm) (ε,Lmol^−1^cm^−1^)
PCHD	256 (10,680)	360, 380 (15,680,15,310)
[Cu(PCHD)_2_]ClO_4_.H_2_O	260 (31,700)	398 (43,500)
PyCHD	260 (32,480)	425 (20,468)
[Cu(PyCHD)_2_]ClO_4_.H_2_O	295 (25,830)	435 (4802)

#### Mass spectrometry

The mass spectra of PCHD and PyCHD given in Additional file 1: Fig. S5 show the molecular ion peak at 170.08 and 171.34 a.m.u., respectively, confirming their formula weights based on their elemental analyses (F.W. 170.17 and 171.16). The mass fragmentation pattern, presented in Additional file 1: Schemes S1 and S2, supported the proposed ligands` structure. The mass spectra of [Cu(PCHD)_2_]ClO_4_.H_2_O and [Cu(PyCHD)_2_]ClO_4_.H_2_O, Additional file 1: Fig. S5 display a well-defined parent peak at m/z 521.03 and 523.10, respectively, in agreement with the molecular weights determined from the postulated chemical formula of the complexes. Suggested fragmentation is summarized in Schemes (Additional file 1: S3 and S4).

#### X-ray photoemission spectroscopy (XPS)

**Fig. 2:**
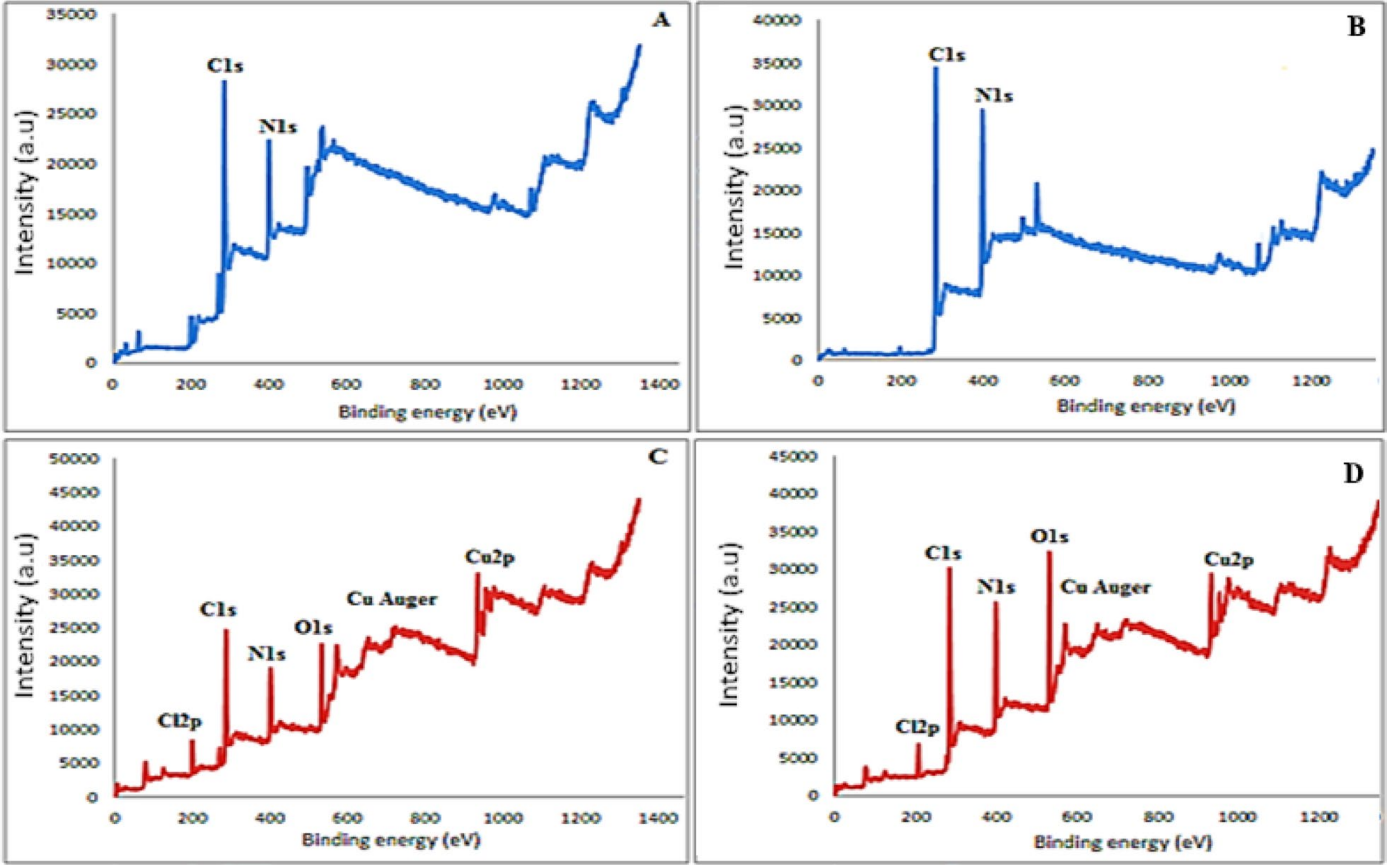
Survey spectra of XPS for (**A**) PCHD, (B) PyCHD, (**C**) [Cu(PCHD)_2_]ClO_4_. H_2_O, and (**D**) [Cu(PyCHD)_2_]ClO_4_.H_2_O

**Fig. 3:**
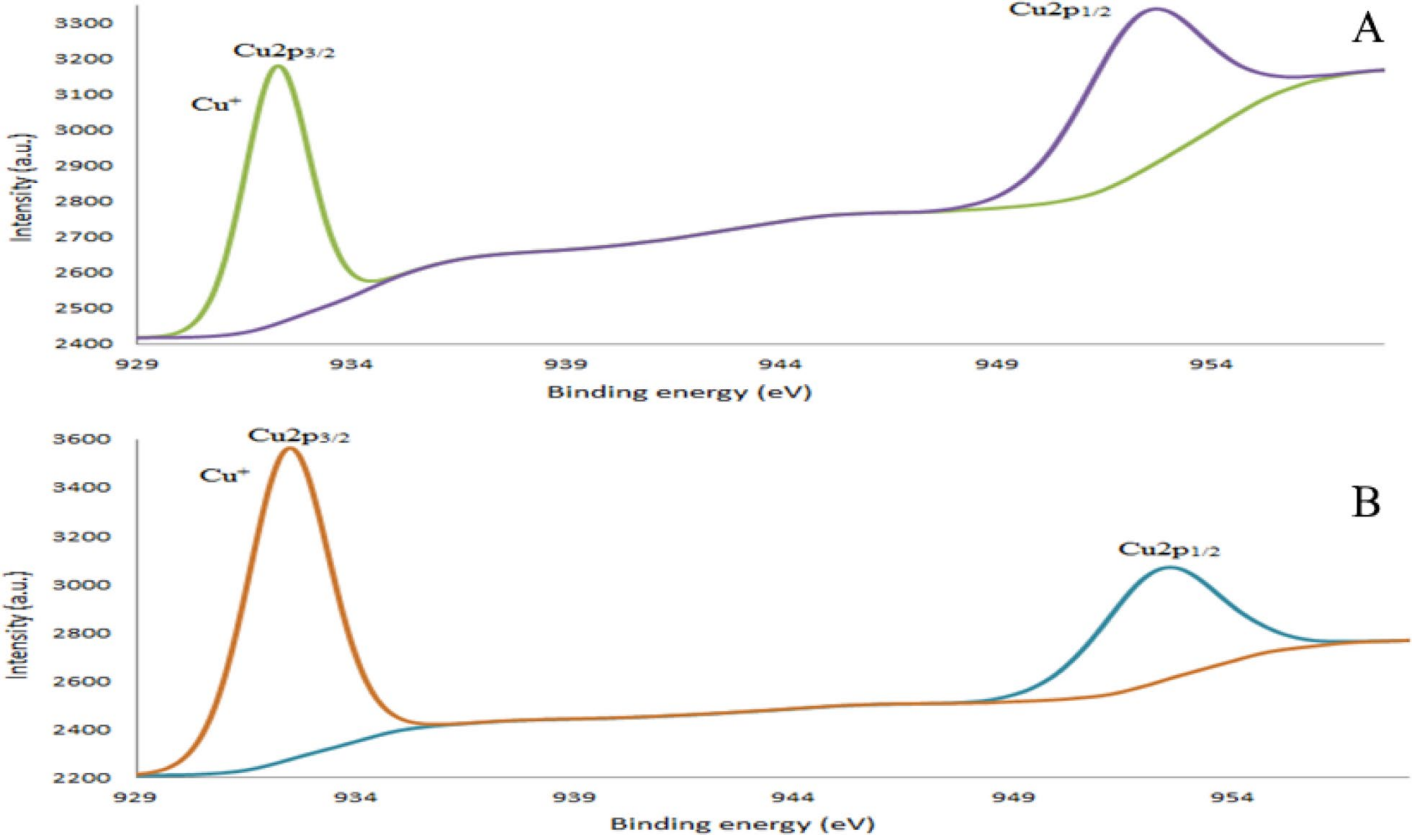
Cu2p XPS spectra for: (**A**) [Cu(PCHD)_2_]ClO_4_.H_2_O, and (**B**) [Cu(PyCHD)_2_]ClO_4_.H_2_O

XPS is very sensitive even to the small changes in the spatial and electronic structure of the compounds, so it is performed to the synthesized compounds for further understanding of the surface elemental compositions and oxidation states of the copper in each complex. The ligands contain C and N atoms while their complexes contain C and N atoms originated from the ligands, in addition to Cu ions, Cl and O originated from the corresponding hydrated copper(II) perchlorate. The wide scan XPS spectra of the free ligands and their copper complexes are shown in Figs. 2, 3 and Additional file 1: Fig. S6–8 the data collected at 1350 eV for Cu(2p_3/2_), Cu(2p_1/2_), C1s, O1s, N1s, and Cl (2p_3/2_), Cl (2p_1/2_) are given in Table 4. Atomic compositions for each sample were calculated using narrow-scan peak areas and the appropriate sensitivity factors for each element. The individual contributions within the high-resolution spectra are reported in Table 4. The high-resolution C1s spectra characterized by contributions at 284.08, 284.48, and 285.88 eV in PCHD arising from the C–(C, H), C = C (sp^2^ bonded carbons), C–C (sp^3^ bonded carbons), C–N, and C ≡ N. The weakly pronounced broad peak at 290.48 eV for PCHD arises from π– π* shake-up transition. An additional peak in PyCHD spectrum appeared at 288.07 eV, corresponding to the C = N of the pyridinyl ring. The deconvolution of N1s peak shows peaks at 398.39 eV attributable to pyridinyl C = N confirming the structure of PyCHD. The spectra of copper complexes exhibit five peaks corresponds to B.E. of C1s, N1s, O1s, Cl2p, and Cu2p, at 285.05, 399.97, 532.03, 198.74, and 933.32 eV for [Cu(PCHD)_2_]ClO_4_.H_2_O, and at 285.43, 399.46, 532.44, 208.08 and 933.84 eV for [Cu(PyCHD)_2_]ClO_4_.H_2_O. The C1s peaks are not affected appreciably upon complex formation (ΔBE) =  ± 0.04 eV). The increase of N1s binding energy to the extent of ΔBE = 0.46 and 0.13 eV for [Cu(PCHD)_2_]ClO_4_.H_2_O and [Cu(PyCHD)_2_]ClO_4_.H_2_O, respectively, supports its bonding to the copper ions via the NH group. The Cu–N bonding was established by the NH and N–C peak shift (∆BE =1.3 and 0.22 eV), upon complexation of PCHD and PyCHD, respectively. Regarding the binding energies of copper in [Cu(PCHD)_2_]ClO_4_.H_2_O, two main peaks are shown at 932.24, 952.39 eV (∆BE = 20.15),corresponding to main characteristic doublets copper 2p_3/2_ and 2p_1/2_ for Cu(I) species [43]. Similarly, the spectrum of [Cu(PyCHD)_2_]ClO_4_.H_2_O shows two peaks at 932.47 and 952.37eV (∆BE = 19.9 eV) characteristic of copper 2p3/2 and 2p1/2 peaks, respectively, indexed to the Cu(I) oxidation state [43, 44]. XPS spectra of Cl2p core levels show bands of (Cl 2p_3/2_ at 199.6 eV, Cl 2p_1/2_ at 197.96 eV) in case of [Cu(PCHD)_2_]ClO_4_.H_2_O and (Cl 2p_3/2_ = 208.7 eV, Cl 2p_1/2_ = 207.11 eV) and for [Cu(PyCHD)_2_]ClO_4_.H_2_O, with peak separation (∆BE = 1.64 and 1.59 eV), respectively, corresponding to the perchlorate group [45].

**Table 4:** XPS data of the free ligands and their copper complexes

Core level	PCHD	Cu(PCHD)_2_]ClO_4_.H_2_O	PyCHD	[Cu(PyCHD)_2_]ClO_4_.H_2_O
	BE	BE	BE	BE
C1s	284.08	–	–	–
C1s a	284.48	284.28	284.40	285.74
C1s b	285.88	285.72	285.51	287.63
C1s c	290.48	287.23	288.07	290.38
N1s	399.15	399.09	399.49	400.01
N1s a	400.56	400.42	400.39	399.44
N1s b	402.14	401.62	402.12	399.71
N1s c	403.77	–	401.44	–
N1s f	397.96	–	398.22	–
N1s h	–	–	403.56	–
O1s	–	531.25	–	532.94
O1s a	–	532.02	–	531.6
O1s b	–	532.02	–	528.63
Cl2 p a	–	199.60	–	208.70
Cl2 p b	–	196.27	–	204.73
Cl2 p c	–	207.10	–	207.11
Cl2 p d	–	201.12	–	–
Cu2p a	–	932.24	–	952.37
Cu2p b	–	952.39	–	932.47

#### Cyclic voltammetry

The redox properties of PCHD, and its copper(I) complex are investigated using cyclic voltammetry (CV), Additional file 1: Figs. S9, 10. The cyclic voltammograms are measured in the potential range +3000 mV to −3000 mV. The electrochemical data of the PCHD and [Cu(PCHD)_2_]ClO_4_.H_2_O are collected in Table 5. [Cu(PCHD)_2_]ClO_4_.H_2_O displayed one redox wave in the 0 to +3000 mV region, Additional file 1: Figs. S9, 10, which is assigned to the Cu(I)/Cu(II) redox couple (~860 mV, vs. Ag/Ag^+^) [46]. This wave is described as a quasi-reversible electron transfer because its peak-to-peak separation ($\Delta$ *E*_p_) is shifted with the scan rate, and the peak current (*I*_p_) varies linearly with the square root of the scan rate Additional file 1: Fig. S11 [47]. The cyclic voltammograms of PCHD ligand did not show any redox wave in the 0 to +3000 mV range. On the other hand, [Cu(PCHD)_2_]ClO_4_.H_2_O exhibited one redox wave in the negative region (0 to—3000 mV), which is assigned to Cu(0)/Cu(I) redox couple (*E*_1/2_ ~ − 660 mV vs Ag/Ag^+^)[48]. Since the peak-to-peak separation ($\Delta$*E*_p_) of this wave is shifted with the scan rate, and the peak current (*I*_p_) varies linearly with the square root of the scan rate, Additional file 1: Fig. S11, this wave is described as a quasi-reversible electrochemical wave, Additional file 1: Fig. S10. Irreversible anodic wave is also observed in this region (~−2300 mV vs Ag/Ag^+^) and it is assigned to the oxidation of the PCHD ligand, since its CV have similar waves (~−2260 mV vs Ag/Ag^+^) but it is slightly shifted toward more negative potential upon metal coordination. This indicates that the coordination of copper increases the electron density on the PCHD ligand. These results reveal the effect of the copper ion on the redox properties of the PCHD ligand. Moreover, the CV of PCHD showed irreversible reduction wave (cathodic) around ~−600 mV, attributed to hydrazo formation [49].

**Table 5:** Cyclic voltammetry data of PCHD ligand and [Cu(PCHD)_2_]ClO_4_.H_2_O^[a]^

Compound	*E*_1/2_ (Δ*E*) (mV)		*E*_*ap*_^[b]^ (mV)	*E*_*cp*_^[b]^ (mV)
	1	2		
PCHD	–	–	2267	−629
CuPCHD	861 (72.4)	− 657 (242)	2323	–
Fc^+^/Fc^[c]^	450	–	–	–

[a] Obtained at a glassy carbon working electrode, platinum wire auxiliary electrode, Ag/AgCl reference electrode and at scan rate 200 mV s^−1^ at room temperature. Supporting electrolyte is 0.1 M TBAPF_6_ in DMSO. The concentration of the compounds is 10^–3^ M. [b] Irreversible anodic and cathodic peaks potentials. [c] Used as standard and measured at the same experimental conditions

### Computational studies

The DFT theoretical methods can provide the electronic properties and geometrical structures of the molecules. Different parameters for example optimization energy, bond lengths, bond angles, quantum parameter such as HOMO and HUMO energies could be calculated from the optimized structure [12].

#### Optimization of the compound geometry

**Fig. 4:**
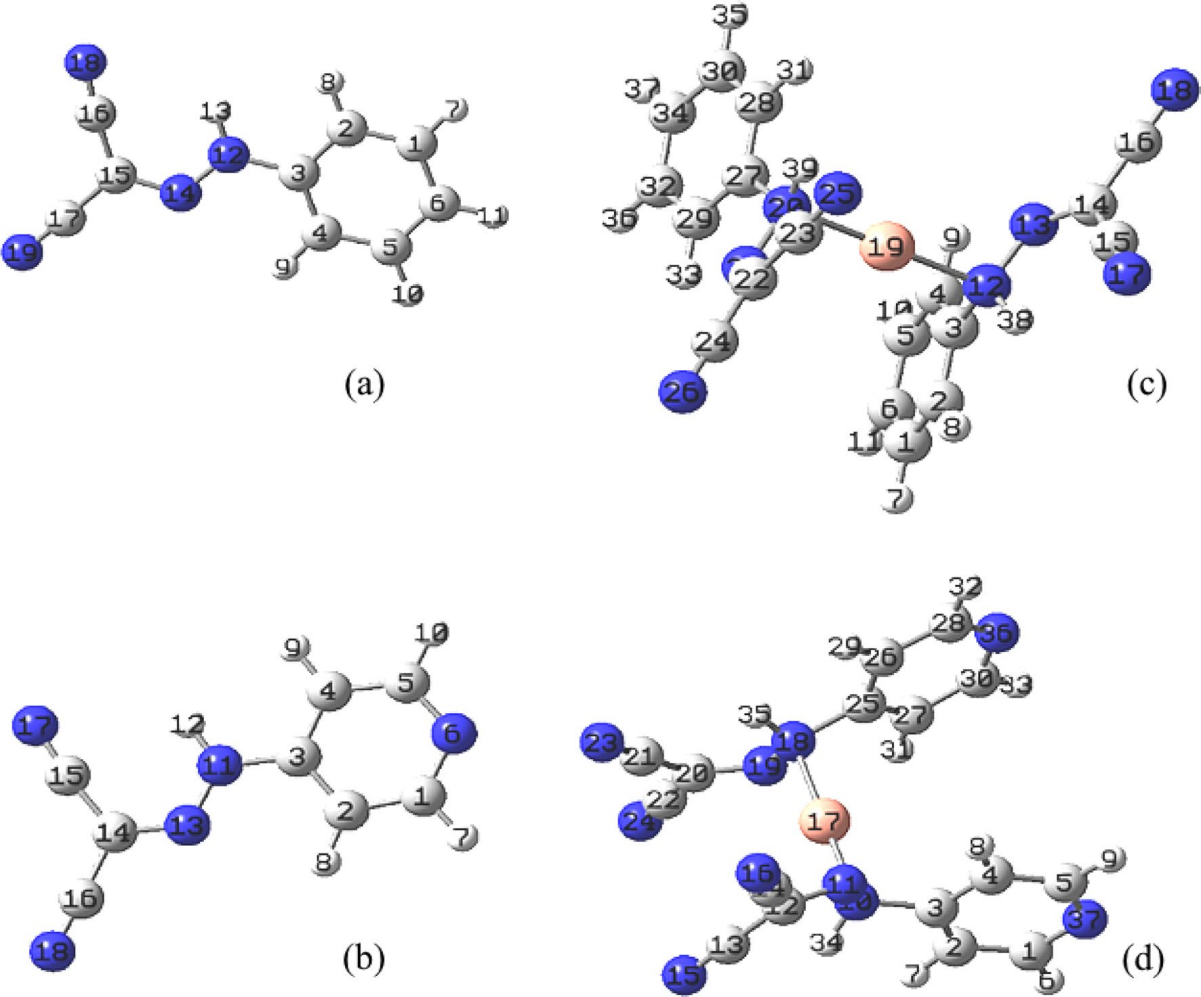
The optimized molecular structure of: (**a**) PCHD, (**b**) PyCHD, (**c**) [Cu(PCHD)_2_]ClO_4_ and (**d**) [Cu(PyCHD)_2_]ClO_4_

The best fitted structural forms for PCHD, PyCHD ligands and their Cu(I)complexes Fig. 4, were established to maintain the bonding nature sandwiched between the ligands and the copper ions. The optimized molecular structure bond lengths and angles were calculated and tabulated in Additional file 1: Table S1–3. The following observations can be drawn:

1. The bond length of C(3)—N(12), N(12)—H(13), benzene ring bond and C(3)—N(11), N(11)- H(12), pyridine ring of [Cu(PCHD)_2_]ClO_4_.H_2_O and [Cu(PyCHD)_2_]ClO_4_.H_2_O complexes, respectively, are stretched as coordination happens with the N-atoms of hydrazo moiety (–C–N–N–H–)[50].
2. Bond angle values of 179.86, (N(20)—Cu(19)—N(12)) and (179.68 N(10)—Cu(17)—N(18)), for [Cu(PCHD)_2_]ClO_4_.H_2_O and [Cu(PyCHD)_2_]ClO_4_.H_2_O complexes, respectively, confirm the suggested linear structures [51].

#### Molecular electrostatic potential (MEP)

**Fig. 5:**
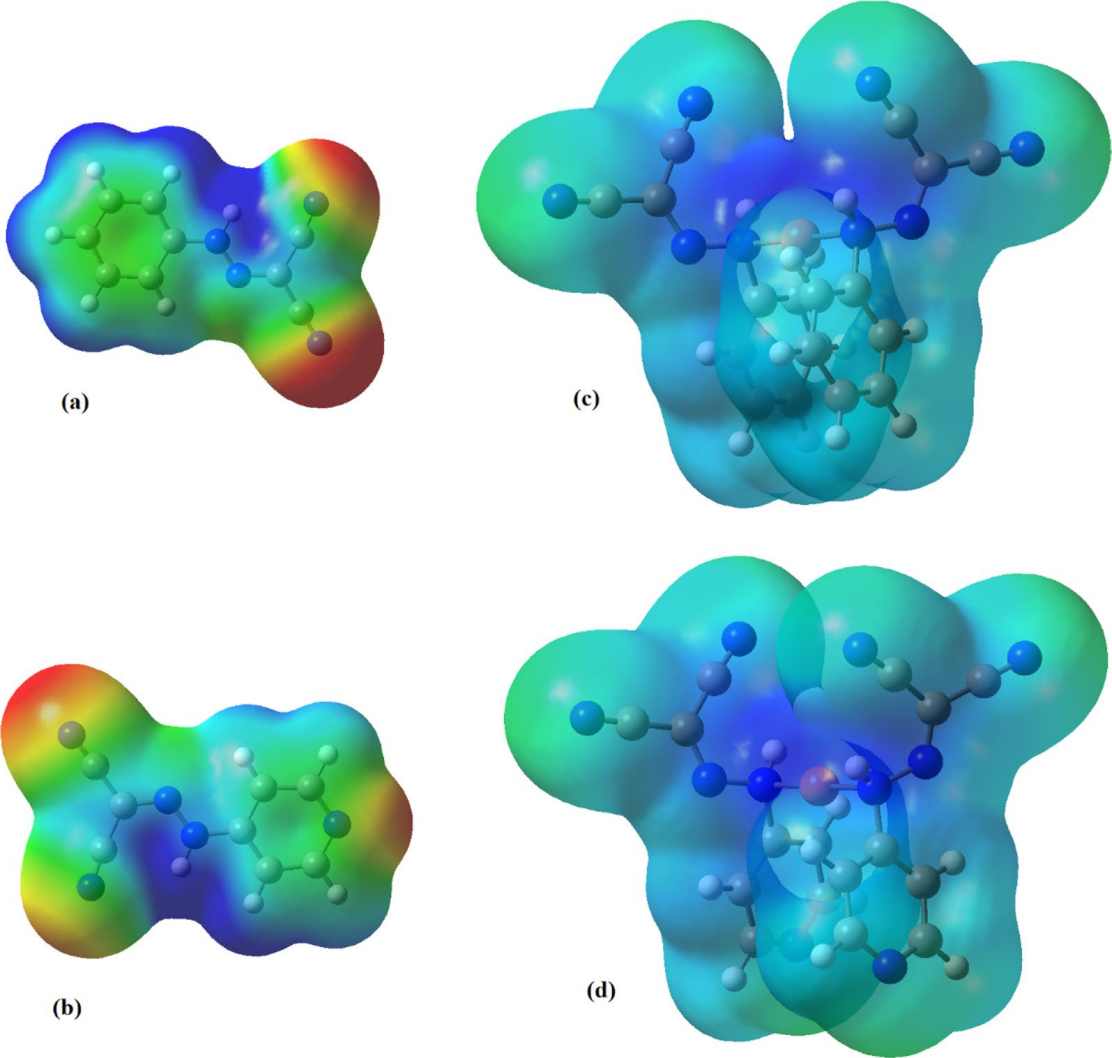
The molecular electrostatic potentials map predicted for: (**a**) PCHD, (**b**) PyCHD, (**c**) [Cu(PCHD)_2_]ClO_4_ and (**d**) [Cu(PyCHD)_2_]ClO_4_

The electron density map through MEP was generated by overlying the Van der Waal’s radii of each atom existing in the compounds so that it revealed the distribution of charge, accordingly, visualize the morphological properties and the reactivity of the molecules. The reactive spots for electrophilic in addition to nucleophilic attack through the chemical reactions can be predicted through maps [52]. MEP diagrams showed the significant reactive regions in the synthesized ligands and their copper complexes Fig. 5. The most electron rich areas in the considered ligands are associated with the nitrogen atom of − C≡N groups as revealed by the red color. Also, this site characterizes the most reactive site as hydrogen acceptor that interacts with the adjacent molecules via electrophilic attack. On the other hands, the blue zone near the proton of the N–H groups revealed the most electron poor sites which are important for the nucleophilic attacks as hydrogen bond donor.

#### Frontier molecular orbitals (FMOs)

**Fig. 6:**
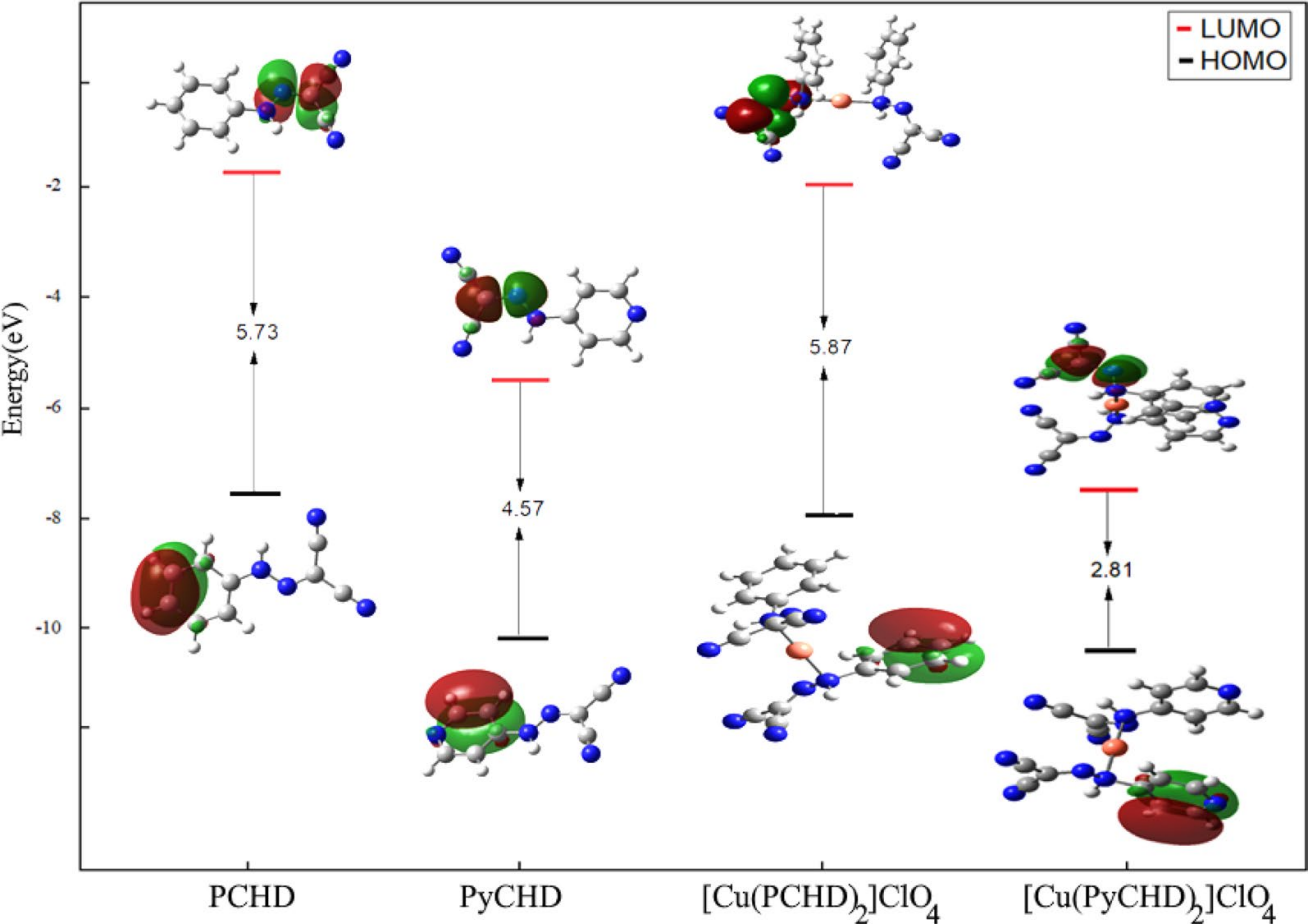
Frontier molecular orbitals diagram (FMOs) of the synthesized ligands and their copper(I) complexes. Negative and positive values of the orbital overlap represented by Green and dark red, respectively

FMOs electronic densities are useful in expecting the reactive positions. The computed energy of formation, HOMO (eV), LUMO (eV) in addition to the dipole moment values were attained for the synthesized ligands and their copper complexes, Table 6. The higher negative values of complexes` formation energy than ligands indicate their higher stability. As shown in Fig. 6, the energy gap (**ΔE**) = E_LUMO_—E_HOMO_ of complexes are smaller than the ligand, that can be attributed to chelation and the effect of copper ions. The small energy gap in both complexes describes the chemical structure, the conformation barriers, the simplicity of charge transfer and electronic transitions inside the complexes [53]. PCHD possessed the highest value of electronegativity at 8.94 eV, whereas [Cu(PyCHD)_2_]ClO_4_.H_2_O complexes possessed the lowest value of electronegativity at 4.65 eV. The whole order of system stability potential (ω), for the synthesized ligands and their copper complexes were noted as follows: (PCHD) > (PyCHD) > ([Cu(PCHD)_2_]ClO_4_.H_2_O) > ([Cu(PyCHD)_2_]ClO_4_.H_2_O. Furthermore, the reduced FMO energy gap observed in the complexes, in comparison to the free ligands, may serve as an indicative factor of their elevated biological activity [10–12], as substantiated in the subsequent Sect. “Biological evaluation”. Additional file 1: Equations S2–7 are used to calculate the quantum parameters of the synthesized compounds, Table 6 [54].

**Table 6:** Quantum chemical properties of the synthesized ligands and their copper complexes

Quantum parameter	PCHD	[Cu(PCHD)_2_]ClO_4_	PyCHD	[Cu(PyCHD)_2_]ClO_4_
E (a.u)	−565.62	−1326.69	−581.69	−1358.73
Dipole moment (Debye)	6.44	4.92	3.46	0.06
E_HOMO_ (eV)	−7.51	−10.11	−7.89	−10.35
E_LUMO_ (eV)	−1.78	−5.54	−2.02	−7.53
ΔE (eV)	5.73	4.57	5.87	2.81
Electronegativity χ (eV)	8.94	4.96	7.82	4.65
ɳ (eV)	1.41	2.93	2.28	2.86
Chemical potentials σ (eV)^−1^	0.71	0.34	0.44	0.35
Pi (eV)	−8.94	−4.96	−7.82	−4.65
Absolute softness S (eV)^−1^	0.36	0.17	0.22	0.17
ω (eV)	28.42	4.19	13.41	3.77
ΔN_Max_ (eV)	6.36	1.69	3.43	1.62
I (eV)	10.34	7.89	10.11	7.51
A (eV)	7.53	2.02	5.54	1.78
Enthalpy (ΔH)	−565.50	−1326.39	−581.55	−1358.45
Free energy (ΔG)	−565.55	−1326.49	−581.60	−1358.54
Entropy (ΔS)	105.67	193.775	105.224	190.26

### Biological evaluation

#### In vitro antimicrobial screening

The synthesized compounds; PCHD, PyCHD, [Cu(PCHD)_2_]ClO_4_.H_2_O and [Cu(PyCHD)_2_]ClO_4_.H_2_O are tested for their antimicrobial activities against *Staphylococcus aureus* and *Bacillus subtilis* as Gram-positive bacteria*, Escherichia coli* and *Proteus vulgaris* as Gram-negative bacteria*,* and *Candida Albicans* and *Aspergillus Fumigatus* as pathogenic fungi, Additional file 1: Figs. S12, 13. The used negative control was DMSO, while the used positive controls were Gentamycin and Ketoconazole as antibacterial and antifungal, respectively. The susceptibility results of these microbial strain panels towards PCHD, PyCHD, [Cu(PCHD)_2_]ClO_4_.H_2_O, and [Cu(PyCHD)_2_]ClO_4_.H_2_O are summarized in Table 7. The growth of the inhibition zone revealed antimicrobial activity against selected organisms. The data showed comparatively larger inhibitory effects of [Cu(PyCHD)_2_]ClO_4_.H_2_O and [Cu(PCHD)_2_]ClO_4_.H_2_O relative to PyCHD or PCHD under the same conditions. The Overtone’s concept and coordination theory clarify the greater efficiency of copper(I) complexes. Based on Overtone’s concept, only lipophilic molecules go through the lipid membrane of cells. Therefore, Lipo-solubility is an important factor that regulates antimicrobial activity. Upon coordination, the associated copper(I) could inhibit cellular enzyme function, or catalyze destructive interactions between cellular constituents[55]. In particular, the investigated compounds show various degree of antibacterial activity, and the activity pattern of these compounds against the growth of most bacteria species is in the order:

**Table 7:** The inhibition diameter zone values (mm ±) for PCHD, PyCHD, [Cu(PCHD)_2_]ClO_4_.H_2_O, and [Cu(PyCHD)_2_]ClO_4_.H_2_O

Compound	Inhibition zone diameter (mm/mg sample)					
	Gram negative		Gram positive		Fungi	
	*E.Coli*	*P.Vulgaris*	*B.Subtilis*	*S.Aureus*	*A. Fumigatus*	*C.Albicans*
**PCHD**	30 ± 0.19	24 ± 0.56	25 ± 0.25	24 ± 0.81	20 ± 0.11	21 ± 0.39
**[Cu(PCHD)**_**2**_**]ClO**_**4**_**.H**_**2**_**O**	39 ± 0.22	34 ± 0.37	30 ± 0.04	32 ± 0.74	40 ± 0.62	35 ± 0.30
**PyCHD**	35 ± 0.64	28 ± 0.55	27 ± 0.29	25 ± 0.22	27 ± 0.22	30 ± 0.50
**[Cu(PyCHD)**_**2**_**]ClO**_**4**_**.H**_**2**_**O**	40 ± 0.23	35 ± 0.26	32 ± 0.36	35 ± 0.41	41 ± 0.37	37 ± 0.09
**Gentamycin**	30 ± 0.15	25 ± 0.32	26 ± 0.41	24 ± 0.19	–	–
**Ketoconazole**	–	–	–	–	17 ± 0.20	20 ± 0.51

[Cu(PyCHD)_2_]ClO_4_.H_2_O > [Cu(PCHD)_2_]ClO_4_.H_2_O > PyCHD > PCHD.

The higher activity of [Cu(PyCHD)]ClO_4_.H_2_O relative to the other compounds is due to nitrogen's capability to create major deviations in the microorganism cells` internal structure, such as nuclear disintegration, mitochondrial function distraction, and cytoplasmic retraction, as well as its resistance to active pathogenic bacteria [56].

In general, the impermeability of the cells or variations in ribosomes are typically responsible for the variation in the activity of the investigated compounds [57]. The results reveal high efficiency of inhibiting the growth of *E.coli and P.vulgaris* relative to *S. aureus and B. subtilis*, Table 7. This variation in activity could be caused by modifications to the cell walls of bacteria, a structure that might be utilised to enhance drug absorption [58]. Noteworthy, [Cu(PyCHD)_2_]ClO_4_.H_2_O is found to be a powerful antifungal agent, towards both *Aspergillus fumigatus* and *Candida albicans* fungi. While PCHD, PyCHD, and [Cu(PCHD)_2_]ClO_4_.H_2_O complex showed respectable activity. As reviewed in the literature [59], the type of donor atoms, metal ion and structure of the metal complexes may influence their bioactivities. It worth to mention that [Cu(PyCHD)_2_]ClO_4_.H_2_O complex is more effective among all the tested compounds, in the in vitro screening tests. The minimum inhibitory concentrations (MIC) of both ligands and their copper(I) complexes were measured by broth microdilution method, Additional file 1: Table S4. [Cu(PyCHD)_2_]ClO_4_.H_2_O showed better potency against tested microbes than other synthesized compounds, and relative to Gentamycin as antibacterial standard or Ketoconazole as antifungal standard.

#### In vitro cytotoxicity screening

**Fig. 7:**
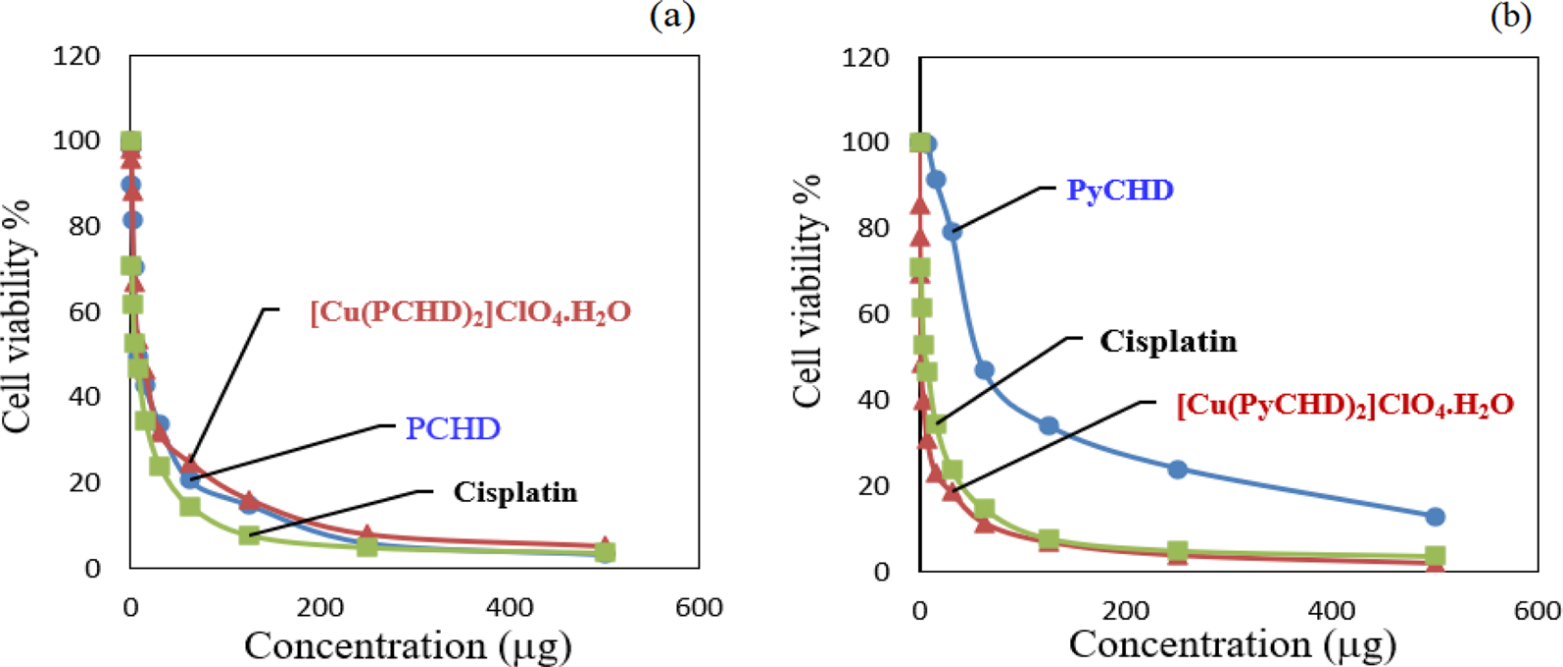
Plot of cell viability % versus concentration of (**a**) PCHD, [Cu(PCHD)_2_]ClO_4_.H_2_O and (**b**) PyCHD, [Cu(PyCHD)_2_]ClO_4_.H_2_O, against MCF-7, compared with Cisplatin

The preliminary cytotoxic activity of the investigated compounds is evaluated versus breast carcinoma human cell lines (MCF7). Different concentrations of PCHD, PyCHD, [Cu(PCHD)_2_]ClO_4_.H_2_O, and [Cu(PyCHD)_2_]ClO_4_.H_2_O are used to calculate the IC_50_ values, expressing the required concentration to inhibit half the culture growth when the cells are treated with the tested compounds for 48 h. Studying the screening results, Table 8 and Fig. 7, some conclusions are accomplished: (i) the values of IC_50_ and the % cell viability revealed that the four explored compounds had a cytotoxicity impact on MCF7, (ii) notable activity enhancement was observed for PyCHD and [Cu(PyCHD)_2_]ClO_4_.H_2_O, (iii) [Cu(PyCHD)_2_]ClO_4_.H_2_O exhibited the greatest activity against breast carcinoma cell lines (MCF-7) with IC_50_, 1.9 ± 0.1 µg/mL, and (iv) in terms of the activities against breast carcinoma (MCF-7), the tested compounds are arranged in comparison to Cisplatin in the following order:

**Table 8:** IC_50_ values (μg/ml) of PCHD, [Cu(PCHD)_2_]ClO_4_.H_2_O, PyCHD, [Cu(PyCHD)_2_]ClO_4_.H_2_O, against MCF-7, compared with Cisplatin, and their binding constant toward wheat DNA

Compound	MCF-7 (breast carcinoma) IC_50_ µg/ml	K_b_(DNA)
Cisplatin	5.71 ± 0.55	––
PCHD	7.75 ± 0.8	3.49 × 10^3^
[Cu(PCHD)_2_]ClO_4_.H_2_O	11.50 ± 0.9	6.60 × 10^3^
PyCHD	59.50 ± 2.9	2.64 × 10^4^
[Cu(PyCHD)_2_]ClO_4_.H_2_O	1.90 ± 0.1	4.75 × 10^4^

[Cu(PyCHD)_2_]ClO_4_.H_2_O > Cisplatin > PCHD > [Cu (PCHD)_2_]ClO_4_.H_2_O > PyCHD. These results demonstrate that the [Cu(PyCHD)_2_]ClO_4_.H_2_O complex can cause cytotoxicity toward diseases and infections mediated by cancer, bacteria, and fungi [60].

#### DNA-binding studies

Generally, DNA binding studies are useful to appreciate the interaction between the synthesized compounds and targeted microbes which may be covalent, intercalation, groove, or phosphodiester backbone binding. Such interaction is greatly affected by the examined compounds nature [61].

**Fig. 8:**
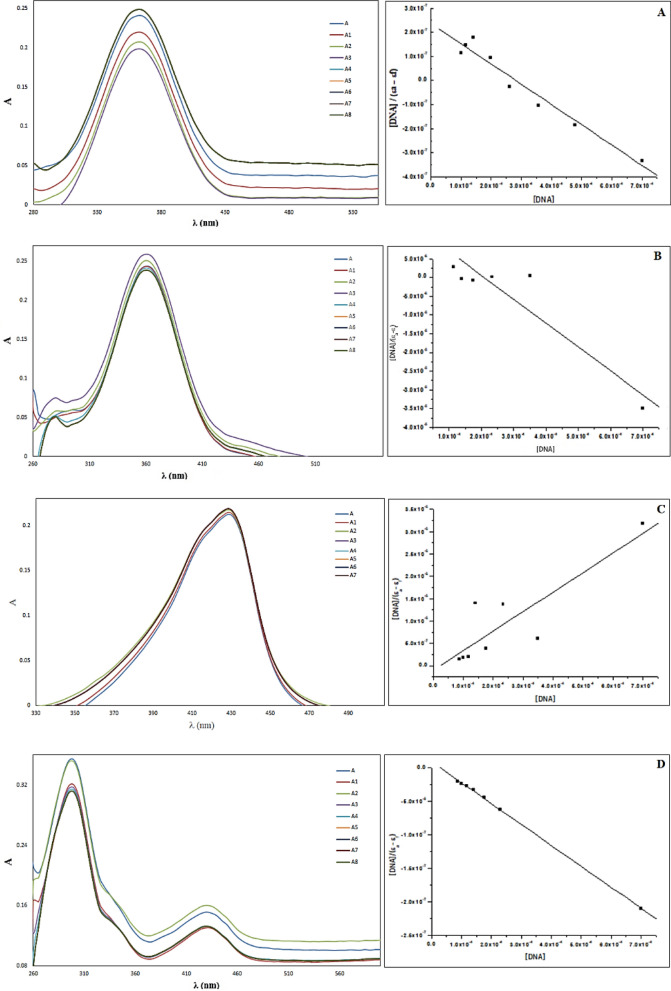
Absorption spectra of 1.00 × 10^–4^ M (**A**) PCHD, (**B**) [Cu(PCHD)_2_]ClO_4_.H_2_O, (**C**) PyCHD, and (**D**) [Cu(PyCHD)_2_]ClO_4_.H_2_O in DMSO solution in the absence and presence of (0–80) µL wheat DNA, and a plot of $\frac{[\mathrm{DNA}]}{(\mathrm{\varepsilon a}-\mathrm{\varepsilon f})}$ vs. [DNA] for the calculation of the intrinsic binding constant, K_b_

The binding capabilities of the PCHD, PyCHD, [Cu(PCHD)_2_]ClO_4_.H_2_O, and [Cu(PyCHD)_2_]ClO_4_.H_2_O are estimated towards wheat DNA, by recording the electronic absorption spectra throughout their interaction [62]. The absorbance measurements were performed by varying the concentrations of DNA (10, 20, 30, 40 up to 90 µL) of 3.5 µM stock wheat DNA, while maintaining the concentration of the ligands or copper complexes (1.0 × 10^–4^ M). The electronic absorption study of PCHD showed an initial decrease in the intensity of the band at 360 nm, within the low concentrations of DNA using volumes from 10 to 30 µL, (hypochromism), Fig. 8A. While upon increasing the amount of DNA up to 40 µL, significant increase in the band intensity (hyperchromism) is observed. No further changes with increasing the DNA up to 80 μL. The electrostatic binding effect or the possibility that PCHD could uncoil DNA’s helix shape and cause additional bases to embed in exposed DNA may be responsible for the hyperchromic effect [29].

For [Cu(PCHD)_2_]ClO_4_.H_2_O, following the intensity change of the band at 361 nm, with increasing the concentration of DNA, an initial increase starts at low concentrations of DNA up to 20 µL (hyperchromism), followed by intensity decrease up to 40 µL (hypochromism), signifying the direct formation of a new complex with double-helical wheat DNA. Figure 8B. However, no substantial shift was detected upon increasing the wheat DNA up to 80 µL, attributed to the π → π* stacking resulted from the interaction between the complex`s phenyl chromophore and DNA base pairs that is steady with the intercalative binding mode [63].

Following the interaction between PyCHD and DNA Fig. 8C, the band at 430 nm exhibited an increase in intensity. The observed hyperchromism suggests the binding mode to wheat DNA, as either external contact and/or uncoil the DNA helix structure by PyCHD, thus resulting in a destabilization of the DNA duplex and made more bases embedding in DNA exposed [29]. However, the DNA’s purine and pyrimidine bases are exposed as a result of the ligand binding to DNA, which often results in an increase in the absorption intensity. The conformation of DNA is somewhat altered by this type of binding [64].

UV spectrum of ([Cu(PyCHD)_2_]ClO_4_.H_2_O / wheat DNA) is shown in Fig. 8D. Increasing the DNA concentrations, the absorption band at 300 nm appeared with. The intercalation way involves a strong π– π* stacking interaction between the aromatic chromophore and the DNA base pairs. Since the extent of the hypochromism in the UV–Vis band is consistent with the strength of intercalative interaction, the investigated compounds interact with wheat DNA quite probably by intercalating the compounds into the DNA base pairs [29, 30].

For each complex, the increment of DNA concentration has been monitored to evaluate the intrinsic binding constant, Table 9, which is observed in the range (3.49 × 10^3^–4.75 × 10^4^). From the calculated K_b_ values, the following points could pick up:

1. The K_b_ value could be interpreted as proof that wheat DNA is strongly bound by the PCHD, PyCHD, and their copper(I) complexes.
2. The binding extent of [Cu(PCHD)_2_]ClO_4_.H_2_O and [Cu(PyCHD)_2_]ClO_4_.H_2_O to the wheat DNA is greater than the PCHD and PyCHD.
3. The presences of heterocyclic N-donor atoms in PyCHD lead to further increase in the binding strength K_b_ due to its additional effect.
4. The order of increasing the binding strength can be given as: [Cu(PyCHD)_2_]ClO_4_.H_2_O > PyCHD > [Cu(PCHD)_2_]ClO_4_.H_2_O > PCHD

**Table 9:** Inhibition constant, energy values of docking simulation of M^pro^ enzyme (PDB: 6WTT) and Estrogen Receptor Alpha Ligand Binding Domain (PDB:6CBZ) with the synthesized compounds

Receptor	Compound	Inhibition constant (µM)	Total binding energy*	Intermolecular energy*	VDW + Hbond + desolvation energy*	Electrostatic energy*	Torsional energy*	Total internal unbound*
**6CBZ**	**PCHD**	64.22	− 5.72	− 6.32	− 6.20	− 0.12	+ 0.60	− 0.21
	**[Cu(PCHD)**_**2**_**]ClO**_**4**_**.H**_**2**_**O**	1.21	− 8.07	− 9.72	− 9.68	− 0.04	+ 1.65	− 0.09
	**PyCHD**	128.59	− 5.31	− 5.90	− 5.79	− 0.11	+ 0.60	− 0.17
	**[Cu(PyCHD)**_**2**_**]ClO**_**4**_**.H**_**2**_**O**	1.90	− 7.81	− 9.45	− 9.46	+ 0.00	+ 1.65	− 0.32
**6WTT**	**PCHD**	108.36	− 5.41	− 6.01	− 5.94	− 0.06	+ 0.60	− 0.28
	**[Cu(PCHD)**_**2**_**]ClO**_**4**_**.H**_**2**_**O**	3.10	− 7.51	− 9.16	− 8.62	− 0.54	+ 1.65	− 0.18
	**PyCHD**	172.67	− 5.13	− 5.73	− 5.65	− 0.08	+ 0.60	− 0.27
	**[Cu(PyCHD)**_**2**_**]ClO**_**4**_**.H**_**2**_**O**	4.90	− 7.24	− 8.89	− 8.32	− 0.57	+ 1.65	+ 0.34

^*^Energy: kcal/mol

#### Molecular docking investigation

**Fig. 9:**
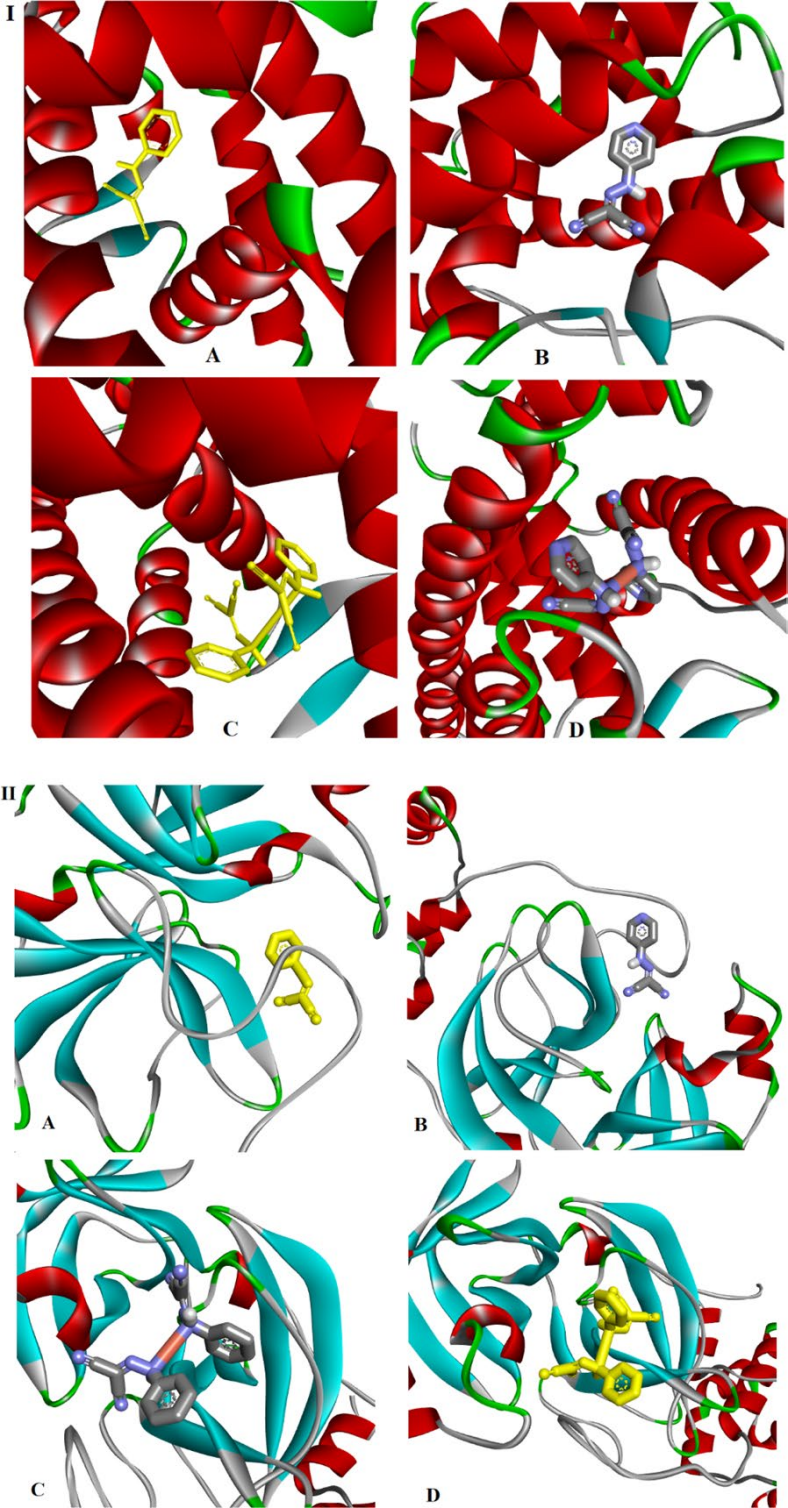
Cartoon representation of (I) Estrogen Receptor Alpha Ligand Binding Domain, and, (II) SARS-CoV M^pro^ protein structures with (**A**) PCHD, (**B**) PyCHD, (**C**) [Cu(PCHD)_2_]ClO_4_.H_2_O and (**D**) [Cu(PyCHD)_2_]ClO_4_.H_2_O

**Fig. 10:**
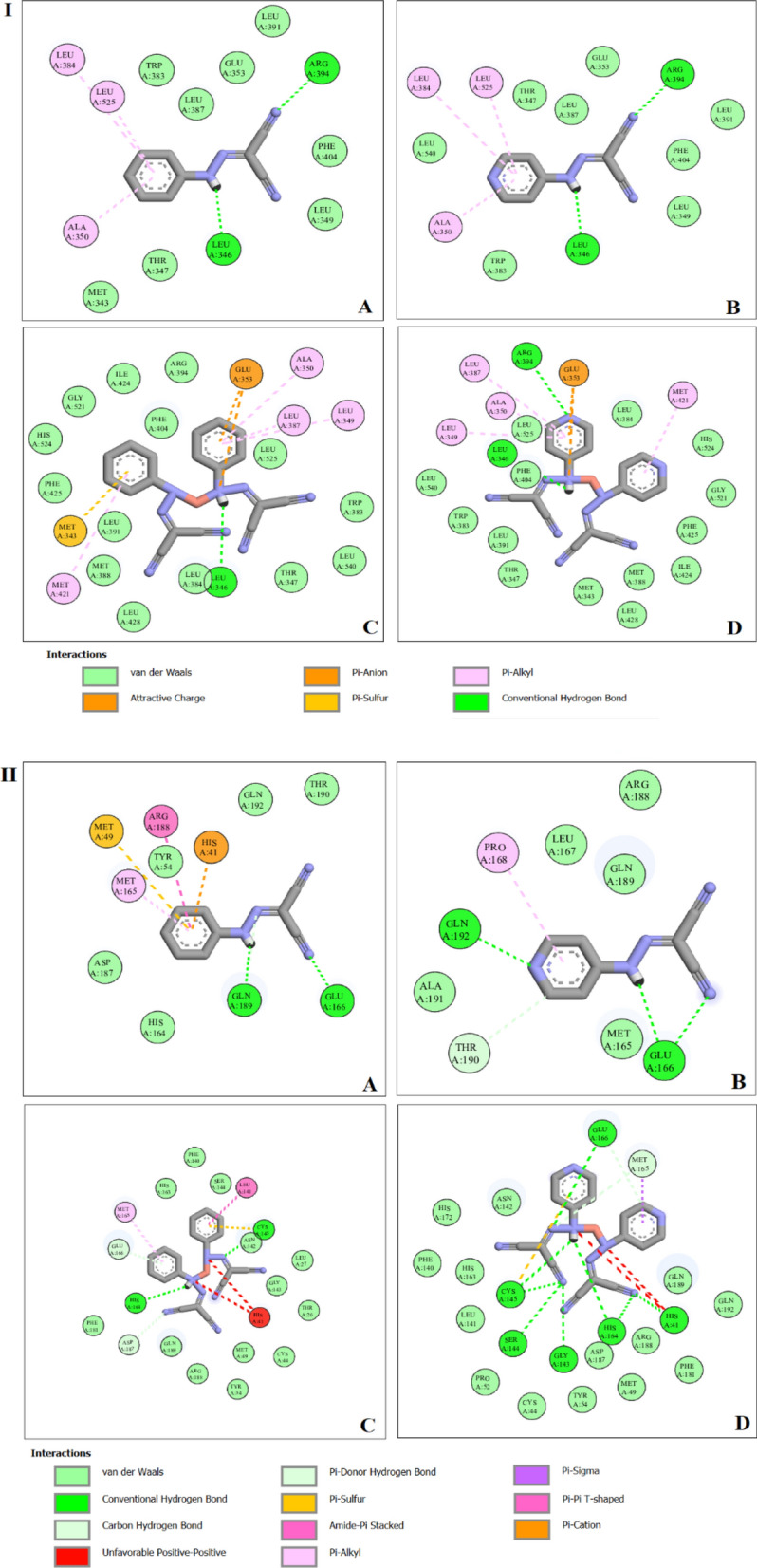
2D mode of binding of: (**A**) PCHD, (**B**) PyCHD, (**C**) [Cu(PCHD)_2_]ClO_4_.H_2_O, and (**D**)[Cu(PyCHD)_2_]ClO_4_.H_2_O with: (I) Estrogen Receptor Alpha Ligand Binding Domain, and, (II) SARS-CoV M^pro^ protein structures

Docking detected protein-active site binding modes. It investigates potential biological activity compounds' mechanisms of action [65]. The essential properties of compounds were discovered in the existence of active sites suitable for hydrogen bonding. This property allows them to be strong inhibitors of protein binding and supports the development of enhanced inhibitory molecules [14, 66]. Ions are interacted with using H-bond, electrostatic, and Van der Waals interactions. Docking simulation produced 50 conformations of the protein-compound complexes. The results demonstrated that the docked compounds recognize the protein’s active site and interact with its key amino acids Fig. 9. The more negative energy, the more stability which means a strong binding between sites and receptors. Therefore, the interaction ability of both copper complexes was higher than their ligands. The results are in agreement with the observations obtained from the in vitro breast cancer cell line assay. To determine which compound is likely to be the most effective inhibitor, we compiled their optimized binding, H-bond, and VDW energies in Table 9. Also, the results were associated with k36 and EST as reference inhibitor ligands Additional file 1: Figs. S14, 15. The interaction between the amino acids of the receptor and k36 proceeds via conventional H-bonding with PHE140, HIS164, GLU166, CYS145 and GLN189, carbon hydrogen bond with HIS172 and π-alkyl interaction with PRO168 and HIS41. The actions of the studied compounds were established to act closely inhibited as k36 and EST as the poses become compatible around the active site. The synthesized ligands and their copper(I) complexes, Additional file 1: Table S5, interact with M^pro^ enzyme (6WTT) and Estrogen Receptor Alpha Ligand Binding Domain (6CBZ) via different types of interactions like, H-bonding, alkyl, π-alkyl and Van der Waals, Fig. 10 [67].

The docking study represented in Additional file 1: Fig. S16 demonstrates the forms of surface interactions of the receptor `s amino acids with ligands and their copper (I) complexes. The green areas of a protein's surface are hydrogen-accepting sites because of their high electron density. The pinkish protein surfaces, however, are severely lacking in electrons. In addition, the hydrophobic interactions shown as VDW and -alkyl bonds, are reflected in the blue surface of the protein residue. And lastly, the brown areas are less hydrophobic [68].

## Conclusion

Synthesis of antimicrobial, antifungal and antitumor redox-active hydrazo containing ligands and their copper(I) complexes were succeeded. The formulation of ligands (PCHD, PyCHD) and their copper(I) complexes ([Cu(PCHD)_2_]ClO_4_.H_2_O, and [Cu(PyCHD)_2_]ClO_4_.H_2_O) were proposed relying on elemental analysis, ^1^H-NMR, mass spectra and conductivity measurements. The complexes are shown to be of Cu(I) as withdrawn from their XPS spectra, ^1^H-NMR spectra, and electrochemistry investigation. Cyclic voltammetry was studied for PCHD and its copper(I) complex, showing the reduction power of the ligand. The *invitro* biological activities revealed that [Cu(PyCHD)]ClO_4_.H_2_O has the higher antimicrobial activity and the greatest activity against breast carcinoma cell lines (MCF-7) with IC_50_, 1.9 ± 0.1 µg/mL relative to the other compounds. Both ligands were bound to DNA through an external contact mechanism. While their corresponding copper(I) complexes were linked through intercalation mechanism. The binding constants for PyCHD and its copper complex were greater than PCHD and its copper complex. Molecular docking was applied to distinguish the potent activity of the ligands and their copper complexes towards SARS-CoV-2 genome protease acceptors M^pro^ (ID:6WTT) and Estrogen Receptor Alpha Ligand Binding Domain (ID:6CBZ), predicting high binding affinity. The synthesized compounds can inhibit COVID-19 viral infection by blocking various active amino acid sides compared to K36 and EST, the standard ligands.

### Supplementary Information

**Additional file 1.** Supplementary file.

## Data Availability

All data are included in the manuscript and supplementary file.
