# Supplementary material for: Synthesis, characterization, DNA binding interactions, DFT calculations, and Covid-19 molecular docking of novel bioactive copper(I) complexes developed via unexpected reduction of azo-hydrazo ligands
Source: BMC Chem. 2023 Nov 20;17(1):159. doi: 10.1186/s13065-023-01086-y (PMC10662581; doi:10.1186/s13065-023-01086-y)
Supplement: Supplementary file 1 — Additional file 1. Supplementary file. [file 13065_2023_1086_MOESM1_ESM.docx]

**Supporting materials**

**Synthesis, characterization, DNA binding interactions, DFT calculations, and Covid-19 molecular docking of novel bioactive copper(I) complexes developed via unexpected reduction of azo-hydrazo ligands**

**Eman Hassan Elsayed^a,^^[[1]](#footnote-1)^, Dhuha Al-Wahaib^b^, Ali El-Dissouky Ali^a^, Beshir A. Abd-El-Nabey^a^, Hemmat A. Elbadawy^a^**

^a^ Chemistry Department, Faculty of Science, Alexandria University, Alexandria, Egypt.

^b^ Chemistry Department, Faculty of Science, Kuwait University, Safat, Kuwait.

**2. Experimental**

### Materials and reagents

Aniline, 4- aminopyridine, malononitrile, sodium nitrite, anhydrous sodium acetate, and copper(II) perchlorate hexahydrate, were purchased from Sigma–Aldrich Chemical Co. Ethanol (ETOH), hydrochloric acid, HCl, and dimethyl sulfoxide, DMSO, were of the analytical reagent grade, and were used without further purification.

### Physical measurements

Melting points were measured using Stuart SMP10 digital melting point apparatus. CHN contents were performed using PerkinElmer, 2400 CHNS Elemental Analyzer 100V, carried out in temperature range from 100 - 1000°C. The copper content in each sample was determined using complexometric titration[1]. HI 8033 HANNA conductometer was used for molar conductivity measurements, at 25$\pm1℃$ for 1.00 x10^-3^ M DMSO solution. Infrared spectra (KBr pellets, 3 mm thickness) were recorded on a Perkin-Elmer Infrared Spectrophotometer (FTIR 1650) in a wavenumber range of 350 - 4000 cm^-1^ at room temperature. UV–visible spectra were recorded as DMSO solution in the concentration range of (1.00x10^-3^–1.00 x10^-6^) M on an automated spectrophotometer UV-Vis Thermo Fischer Scientific Model Evolution 300 ranged from 200 to 1000 nm, at room temperature. Electron impact mass spectrometry (EI-MS) was recorded on direct probe controller inlet part to single quadrupole mass analyzer in the thermo-scientific GCMS model (ISQ LT). Cyclic voltammetry (CV) experiments were carried out using BASi Epsilon 100w potentiostat. A single compartment cell was used, and the electrode system consisted of glassy carbon working electrode, platinum wire auxiliary electrode, and Ag/AgCl reference electrode. The supporting electrolyte TBAPF_6_ was dissolved in DMSO. All of the CV measurements were conducted after purging the solution with nitrogen gas. The concentration of the complexes was 10^−4^ M and prior to any measurements, the potential was calibrated against ferrocene couple (0.44 V Fc^+^/Fc vs. Ag/AgCl reference electrode). X-ray photoelectron spectroscopy (XPS) analyses were performed by K-ALPHA (Thermo Fisher Scientific, USA) with monochromatic X-ray Al K-alpha radiation (energy -10 to 1350 eV) under a vacuum of 10^-9^ mbar with full-spectrum pass energy 200 eV at narrow-spectrum 50 eV. The analysis spot size was 400 μm in diameter. All binding energy values were determined to C1s line originating from adventitious carbon.

### Characteristics of synthesized solids

***C_9_H_6_N_4_ (PCHD)****: yellow solid, Yield: (*5.5*g,* 97*%), m.p= 132±1°C. Anal. Calc. (found): C, 63.52(63.50); H, 3.55(3.52); N, 32.92(32.87), m/z = 170.06 a.m.u (Calc. 170.07). FTIR spectra (KBr, cm^-1^); ν _(NH)_ 3469 (br,m), ν_(-CH-)_ 3060 (w), ν_(C≡N)_ 2232(vs), ν_(C=N)azine_ 1605(s), ν_(C=C)_ 1474 (s). UV-vis. (λ(nm), ε(Lmol^-1^cm^-1^)); (π-π*) 256 (10680), (n- π*) 360(15680), 380(15310). ^1^HNMR (DMSO‑d_6_, δ ppm); (NH),13.07, phenyl group [(H)7.12, (2H)7.34 , J = 8.2 Hz, and (2H)7.39], CH; 1.89.* ^13^C-NMR (*DMSO‑d_6_, δ ppm*) ; 84.249, 111.010, 115.511, 117.172, 126.252, 130.015, 142.791.

***C_8_H_5_N_5_ (PyCHD)****: yellow solid, Yield: (1.30 g, 54 %), m.p= 200±1°C. Anal. Calc. (found): C, 56.09(56.12); H, 2.92(3.11); N, 40.90(41.05), m/z = 171.34 a.m.u (Calc. 171.16). FTIR spectra (KBr, cm^-1^); ν_(NH)_ 3440 (br,m), ν_(-CH-)_ 3079 (w), ν _(C≡N)_ 2215(vs), ν _(C=N)azine_ 1634 (s), ν_(C=N)pyridine_ 1563(w) ν_(C=C)_ 1491 (s). UV-vis. (λ(nm), ε(Lmol^-1^cm^-1^)); (π-π*) 260 (32480), (n- π*) 425(20468). ^1^HNMR (DMSO‑d_6_, δ ppm); [δ 7.36, J = 6.7 Hz,(2H), and (δ 8.33, J = 6.2 Hz,(2H)], CH; 1.89.* ^13^C-NMR (*DMSO‑d_6_, δ ppm*) ; 77.44, 114.28, 116.18, 116.28, 120.32, 121.36, 145.66, 147.47, 150.59, 159.47.

***C_18_H_14_N_8_O_5_ClCu [Cu(PCHD)_2_]ClO_4_.H_2_O****: Yield (0.21 gm, 67 %), m.p > 300°C, Ʌ_m_ = 28.27Ω^-1^cm^-1^mol^-1^. Anal. Calc. (found): C, 41.43(41.39); H, 2.69(2.61); N, 21.18(21.39), Cu, 12.18(12.11), m/z = 521.03 a.m.u (calc. 521.34). FTIR spectra (KBr, cm^-1^); ν _(NH2)_ 3408 (br,w); ν _(C-H)_ 3044(w); ν _(C≡N)_ 2183(vs); ν _(C=N)_, 1640(m); ν _(C=C)_ 1450, ν _(Cu-N)_ 482, ν _(ClO4-)_; 1169(s), 928(w). UV-vis. (λ(nm), ε(Lmol^-1^cm^-1^); (π-π*) 260 (80600); (n- π*)395(42300). ^1^HNMR (DMSO‑d_6_, δ ppm); (NH),14.13, phenyl group [(2H)7.21, (4H)7.29 and (4H)7.40], CH(2H); 1.38.*

***C_18_H_14_N_8_O_5_ClCu [Cu(PyCHD)_2_]ClO_4_.H_2_O****: Yield (0.21 gm, 67 %), m.p > 300°C, Ʌ_m_ = 37.69 Ω^-1^cm^-1^mol^-1^. Anal. Calc. (found): C, 36.69(36.87); H, 2.29(2.53); N, 26.75(27.11), Cu, 12.14(12.27), m/z = 523.10 a.m.u (calc. 523.02). FTIR spectra (KBr, cm^-1^); ν _(NH2)_ 3476 (br,w); ν _(C-H)_ 3060(w); ν _(C≡N)_ 2226(vs); ν _(C=N)azine_, 1605(vs); ν _(C=N)pyridine_ 1560(w); ν _(C=C)_ 1435(vs), ν _(Cu-N)_ 431(m), ν_(ClO4-)_; 1098(vs), 929(vw). UV-vis. (λ(nm), ε(Lmol^-1^cm^-1^)); (π-π*) 260 (80600); (n- π*)395(42300). ^1^HNMR (DMSO‑d_6_, δ ppm);[(4H)6.58, and (4H)7.86], CH(2H); 1.01.*

### Antimicrobial evaluation of the compounds

The microbial activities of the free ligands and their copper complexes were screened against pathogenic bacterial strains *Staphylococcus aureus* (RCMB 010010) and *Bacillus subtilis* (RCMB 015 (1) NRRL B-543) (Gram-positive bacteria), *Escherichia coli* (RCMB (010052) ATCC 25955) and *Proteus vulgaris* (RCMB 004 (1) ATCC 13315) (Gram-negative bacteria) and pathogenic fungi *Candida Albicans* (RCMB 005003 (1) ATCC 10231) and *Aspergillus Fumigatus* (RCMB 002008). The antimicrobial activities of the tested compounds were evaluated using a modified Kirby-Bauer disc diffusion method[2]. The 5 mg/mL stock solutions prepared in DMSO were aseptically diluted two-fold to have solutions of different concentrations. Filter paper disc method[3] and measurements of the inhibition zone diameter (mm) were used to measure the antibacterial and antifungal activity of the test substances. Media with DMSO was used as control. All cultures were routinely maintained on NA (nutrient agar) and incubated at 37 °C. The inoculums of bacteria were performed by growing the culture in NA broth at 37°C for overnight. Approximately 0.1 mL of diluted bacterial or fungal culture suspension was spread uniformly on NA plates. Solutions of the test compounds and reference drugs were prepared by dissolving 10 mg of the mixture in 10 mL DMSO. A 100 µL volume of each sample was pipetted into a hole made in the center of the agar. Sterile 8 mm discs (Himedia Pvt. Ltd.) were impregnated with test compounds. The disc was placed on a plate having one control disc impregnated with solvent. The plates were incubated at 37°C for 18-48 h. Standard discs of Gentamycin (Antibacterial agents; 4 µg / disc) and Ketoconazole (Antifungal agent; 100 µg / disc) served as positive controls for antimicrobial activity while filter discs impregnated with 10 µL of solvent DMSO were used as a negative control.

### Cytotoxicity evaluation of the compounds

A fresh medium containing different concentrations of the test sample was added after 24 h of seeding. Serial twofold dilutions of the tested chemical compound were added to confluent cell monolayers dispensed into 96-well, flat-bottomed micro-liter plates (Falcon, Nu, USA) using a multichannel pipette. The microliter plates were incubated at 37 °C in a humidified incubator with 5% CO_2_ for 24 hrs, and three wells were used for each concentration of the test samples. Control cells were incubated without a test sample and with or without DMSO. The small percentage of DMSO present in the wells (maximum 0.1) does not affect the experiment. After incubation of the cells for 24 h at 37 °C, various concentrations of the sample (50, 25, 12.5, 6.25, 3.125, and 1.56 μg) were added, and the incubation was continued for 48 h; viable cells were determined by a colorimetric method. In brief, after the end of the incubation period, media were aspirated, and crystal violet solution (1%) was added to each well for at least 30 min. The stain was removed, and the plates were rinsed using tap water until all excess stain is removed. Glacial acetic acid (30%) was added to all wells and mixed thoroughly. The absorbance of the plates was measured after gently shaking on a Microplate reader (TECAN, Inc.) using a test wavelength of 490 nm. All results were corrected for background absorbance detected in wells without added stain, and the treated samples were compared with the cell control. The experiments were carried out in triplicate.

### DNA-binding studies by UV-absorption spectroscopy

The interaction of the ligands and their copper complexes with DNA was recorded utilizing UV spectroscopy in the wavelength range of 200 - 1000 nm. The absorbance measurements were performed by varying the concentrations of DNA (10, 20, 30, 40 up to 90 µL)

of 3.5 µM (2.00 ml) stock wheat DNA, while maintaining the concentration of the ligands or copper complexes (1.00×10^-4^ M). The interaction of the ligands or copper complexes with DNA and the intrinsic binding constant (k_b_) of the complexes were determined by using equation 1.

$\frac{[DNA]}{(\varepsilon_{a}-\varepsilon_{f})}=\frac{[DNA]}{(\varepsilon_{b}-\varepsilon_{f})}+\frac{1}{[K_{b}\left( \varepsilon_{b}-\varepsilon_{f} \right)]}$ (S1)

where [DNA] is the concentration of DNA in base pairs, Ԑ_a_ is the apparent coefficient of A_obsd_/[complex], Ԑ_f_, and Ԑ_b_ are extinction coefficients of the free and fully bound forms of the complexes, respectively. A plot of [DNA]/(Ԑ_a_– Ԑ_f_) versus [DNA] gave a slope of 1/(Ԑ_b_ – Ԑ_f_) and intercept equals 1/K_b_(Ԑ_b_– Ԑ_f_), K_b_ is the ratio of a slope to intercept. In order to quantitatively compare the binding strength of the complexes to wheat DNA, the intrinsic binding constants K_b_ of the ligands and their copper complexes were calculated using the UV spectral data of the ligands and their complexes recorded for a constant concentration in the absence and in presence of DNA for diverse mixing ratios (r = [compound]/ [DNA]) by monitoring the changes in the absorbance at the corresponding λ_max_ to ligands and copper complexes, with increasing concentrations of DNA[4].

**Figures:**


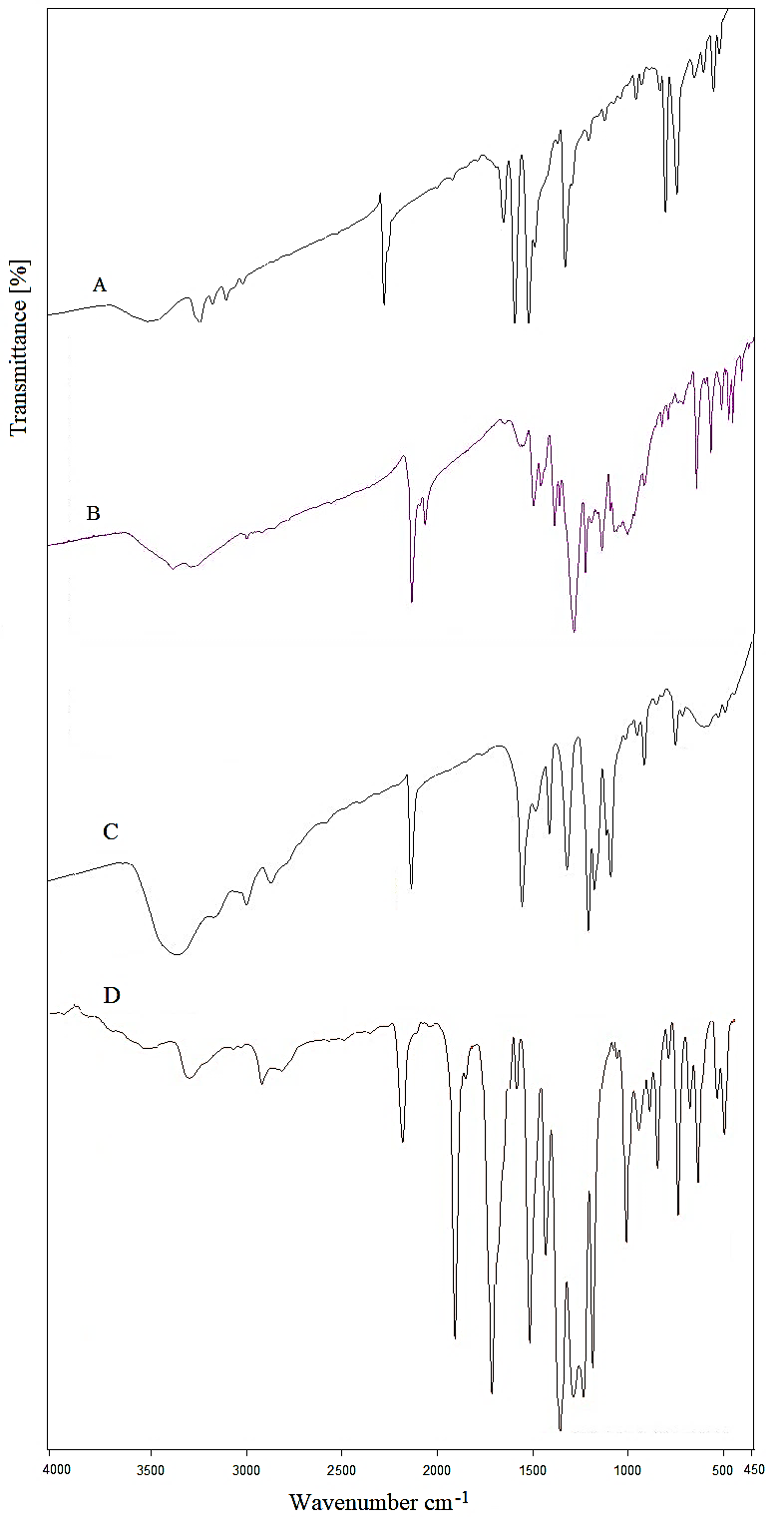


**Fig. (S1): Infrared spectra of (A) PCHD ,(B) [Cu(PCHD)_2_]ClO_4_.H_2_O, (C) PyCHD and (D) [Cu(PyCHD)_2_]ClO_4_.H_2_O**

**
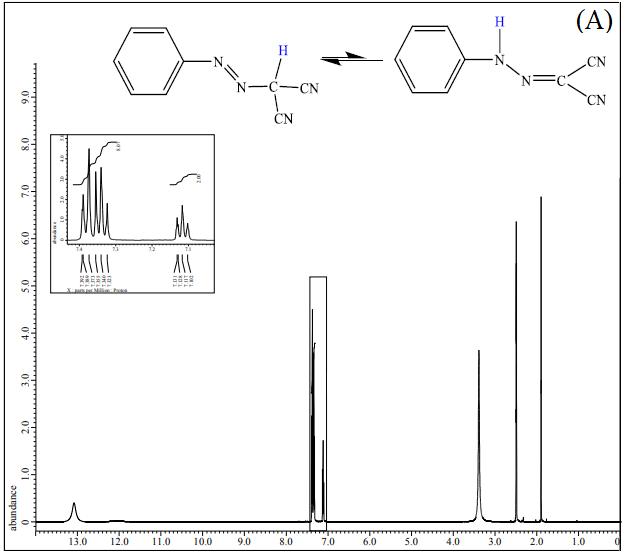
**

**
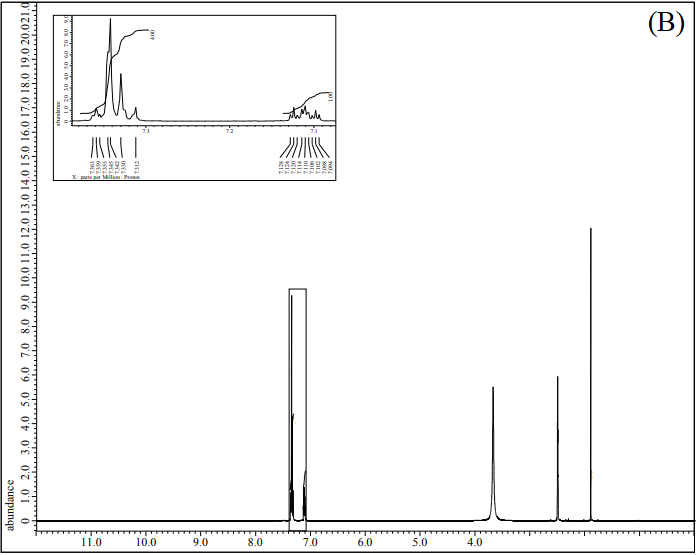
**

**
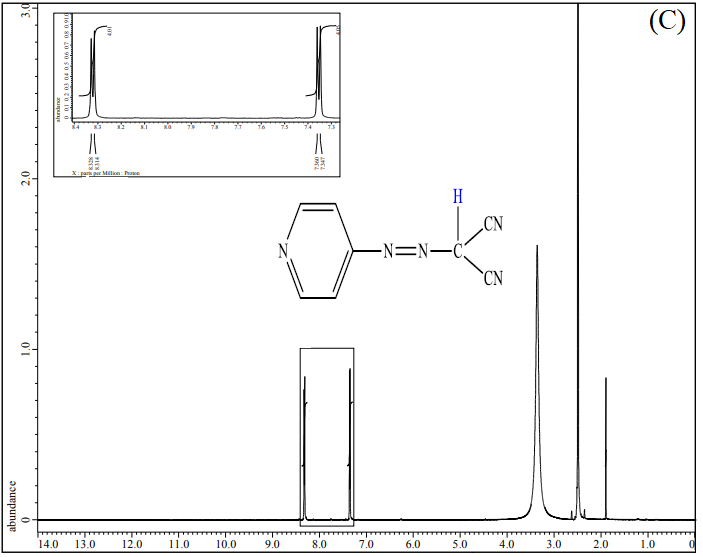
**

**Fig. (S2): ^1^HNMR spectrum of (A) phenylcarbonohydrazonoyl dicyanide ligand (****PCHD) in d_6_-DMSO, (B) in presence of D_2_O and (C) pyridin-4-ylcarbonohydrazonoyl dicyanide (****PyCHD) in d_6_-DMSO.**

**
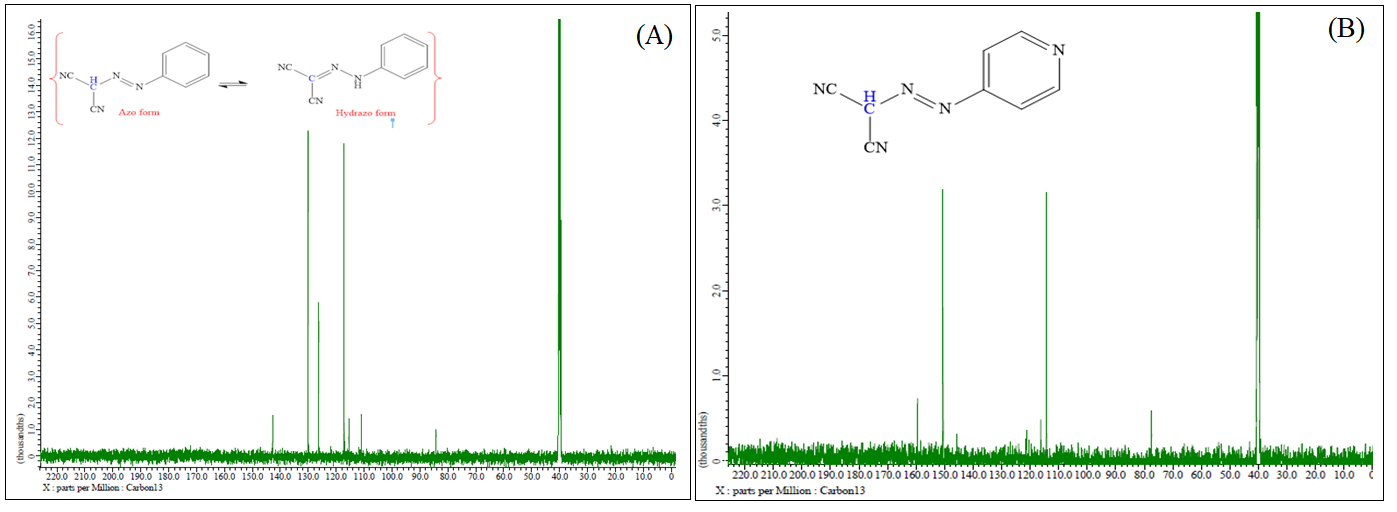
**

**Fig. (S3): ^13^C-NMR spectrum of (A) phenylcarbonohydrazonoyl dicyanide ligand (PCHD) in d_6_-DMSO, and (B) pyridin-4-ylcarbonohydrazonoyl dicyanide (PyCHD) in d_6_-DMSO.**


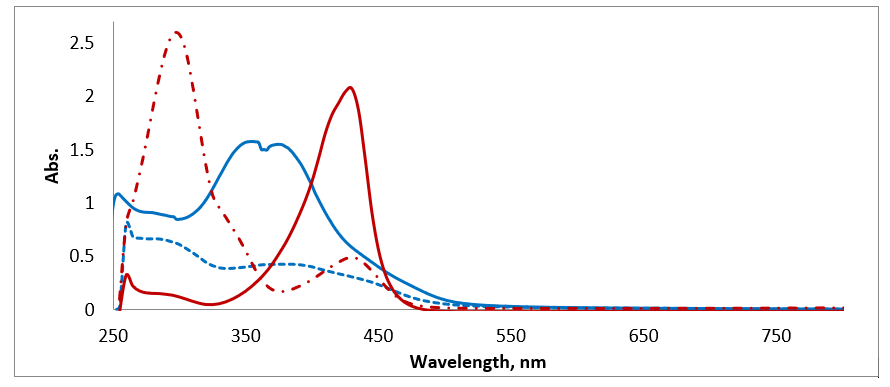


**Fig. (S4): UV-Vis spectra of 1 x 10^-4^ M solution of (―) PCHD, (---) [Cu(PCHD)_2_]ClO_4_.H_2_O, (―) PyCHD and (-.-.-) [Cu(PyCHD)_2_]ClO_4_.H_2_O in DMSO.**

***
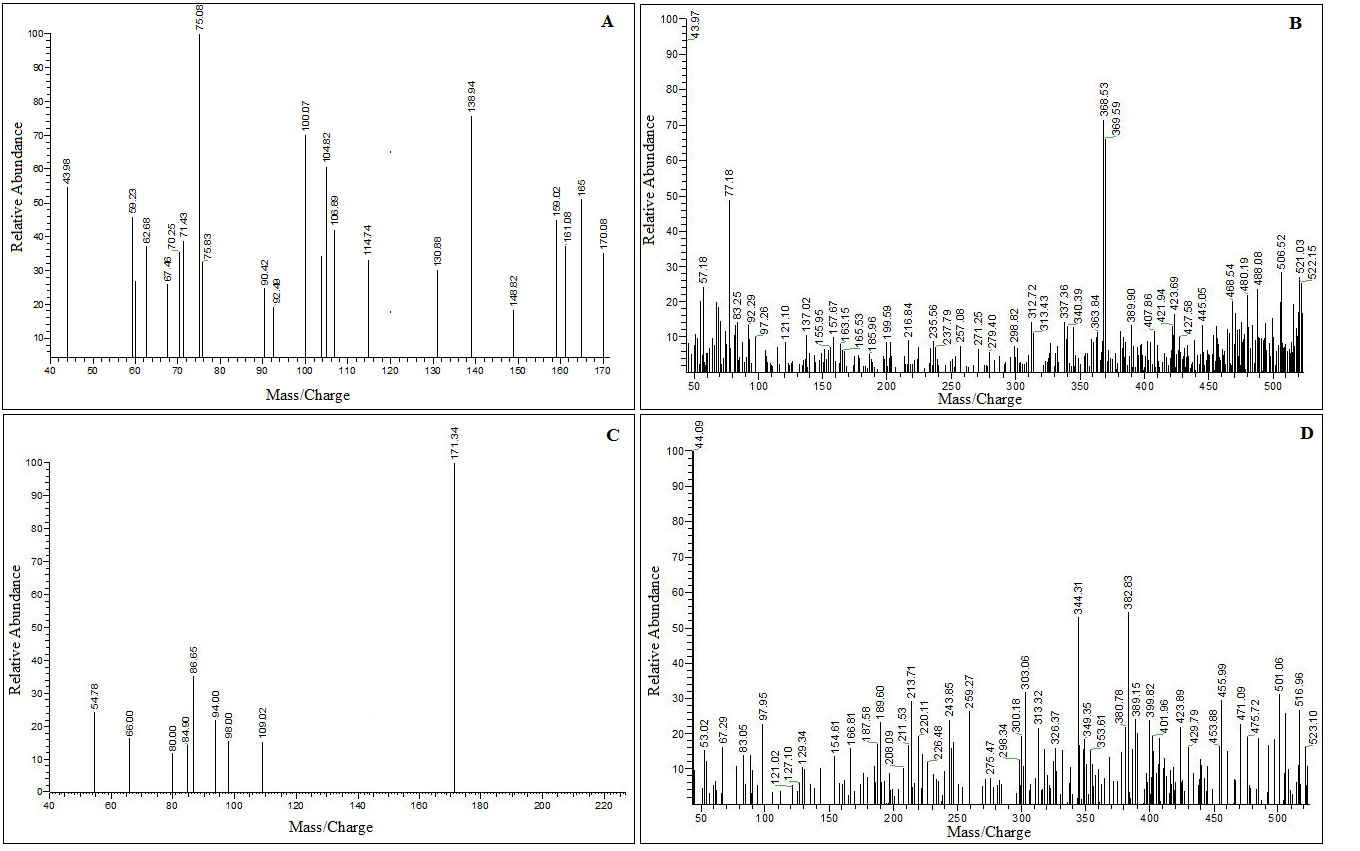
***

**Fig. (S5): Mass spectrum of (A) phenylcarbonohydrazonoyl dicyanide ligand (PCHD), (B) [Cu(PCHD)_2_]ClO_4_.H_2_O, (C) pyridin-4-ylcarbonohydrazonoyl dicyanide ligand (PyCHD)**, **and (D) [Cu(PyCHD)_2_]ClO_4_.H_2_O.**


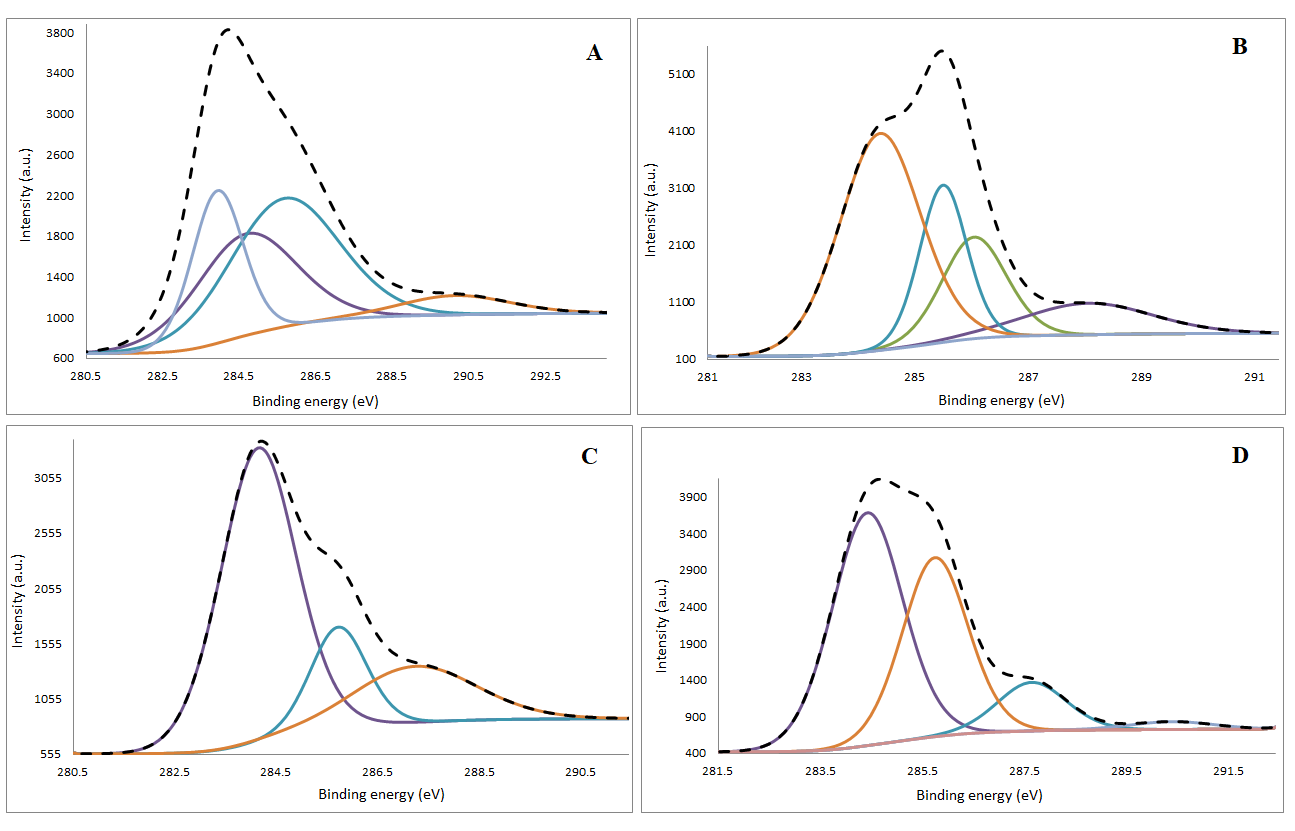


**Fig. (S6): High-resolution C1s XPS spectra for (A) PCHD, (B) PyCHD , (C) [Cu(PCHD)_2_]ClO_4_. H_2_O, and (D) [Cu(PyCHD)_2_]ClO_4_.H_2_O**


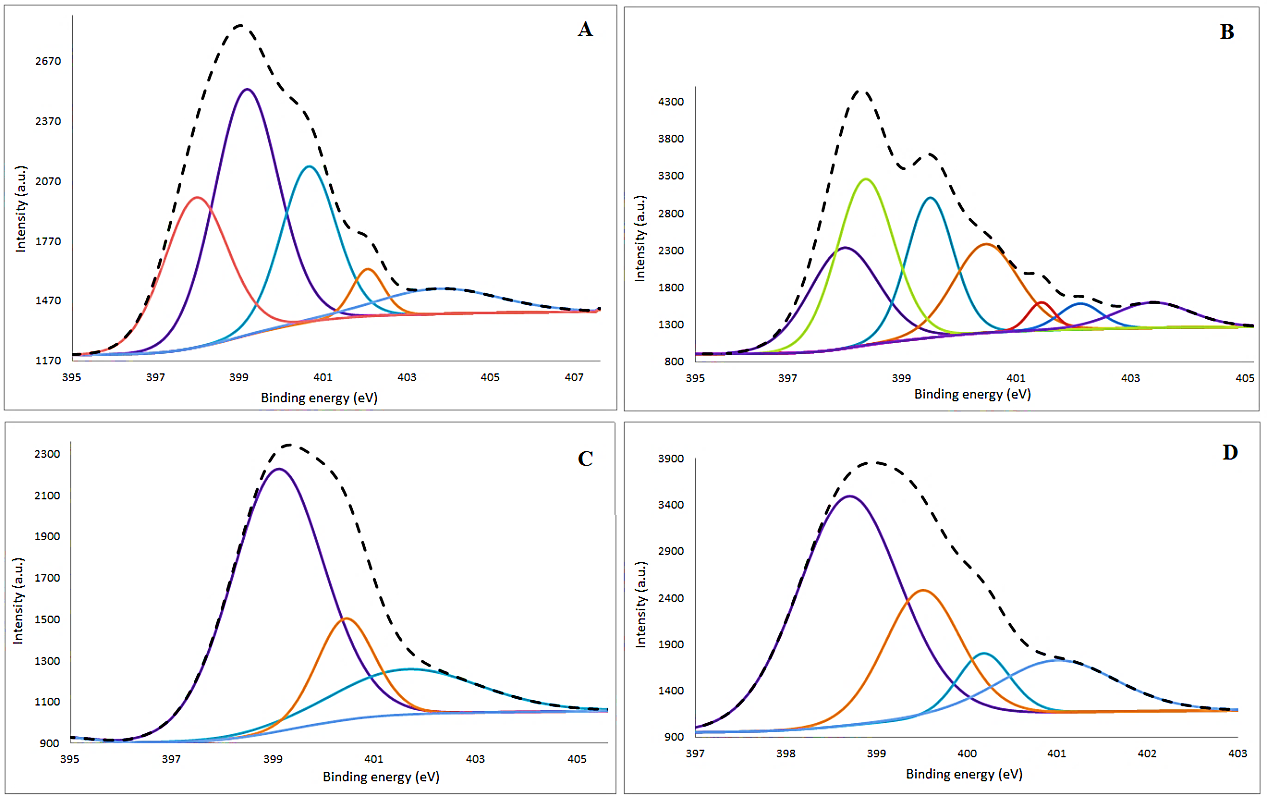


**Fig. (S7): High-resolution N1s XPS spectra for (A) PCHD , (B) PyCHD , (C) [Cu(PCHD)_2_]ClO_4_. H_2_O, and (D) [Cu(PyCHD)_2_]ClO_4_.H_2_O.**


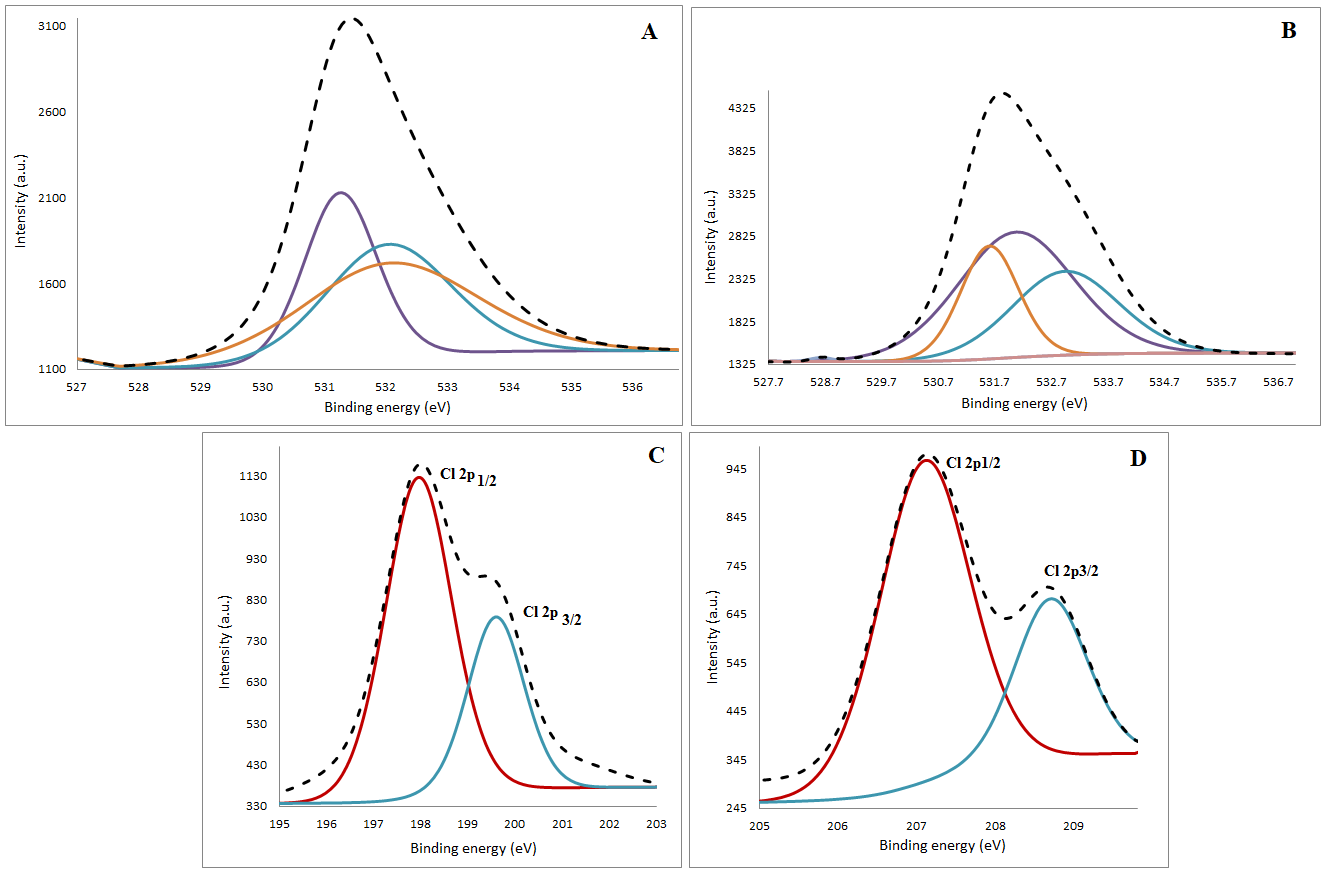


**Fig. (S8): High-resolution O2p & Cl2p XPS spectra for (A&C) [Cu(PCHD)_2_]ClO_4_.H_2_O, and (B&D) [Cu(PyCHD)_2_]ClO_4_.H_2_O, respectively.**


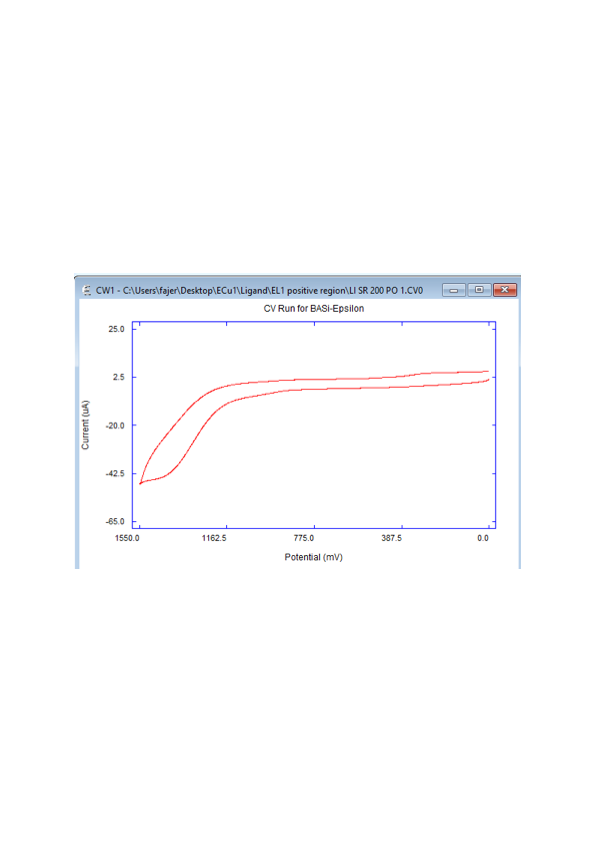


PCHD


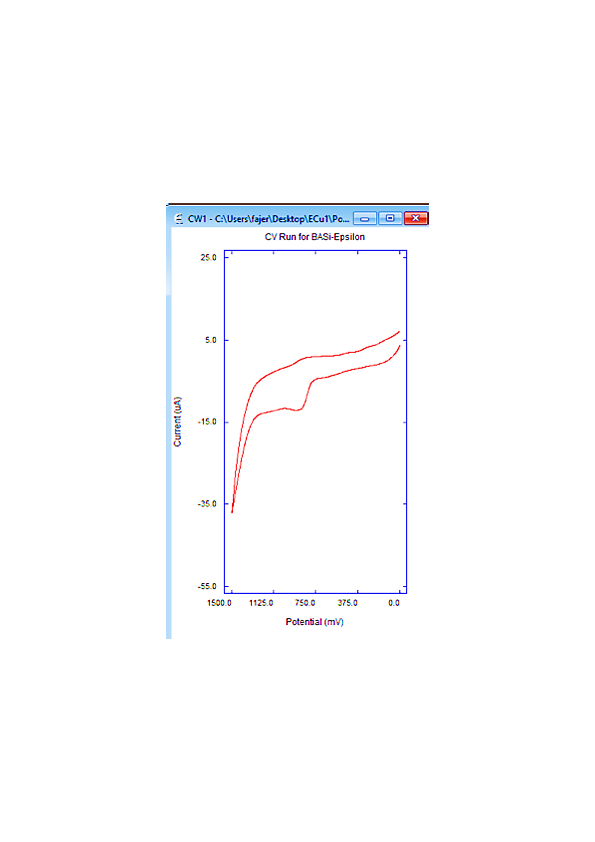


[CuPCHD)_2_]ClO_4_

**Fig. (S9): Cyclic voltammograms in the positive region of PCHD ligand and its copper complex,[Cu(PCHD)_2_]ClO_4_, at a glassy carbon working electrode, platinum wire auxiliary electrode, Ag/AgCl reference electrode and at scan rate 200 mV s^-1^ at room temperature. Supporting electrolyte is 0.1 M TBAPF_6_ in DMSO. The concentration of the compounds are 10^-3^ M.**


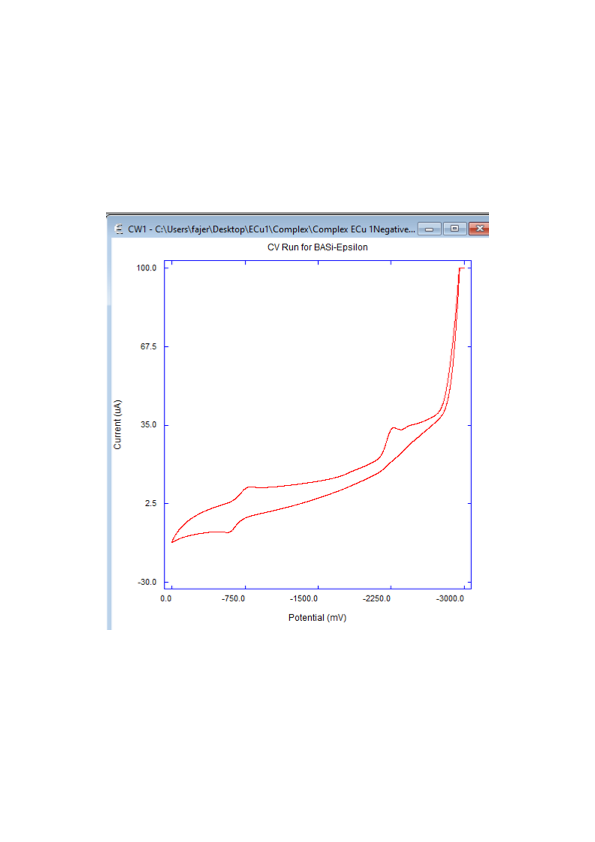


[CuPCHD)_2_]ClO_4_


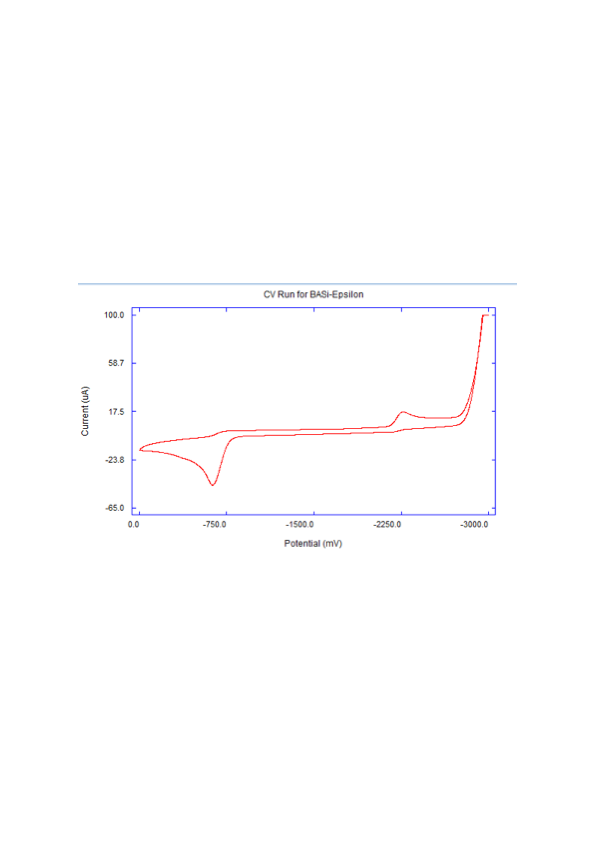


PCHD

**Fig. (S10): Cyclic voltammograms in the negative region of PCHD ligand and its copper complex, [Cu(PCHD)_2_]ClO_4_, obtained at a glassy carbon working electrode, platinum wire auxiliary electrode, Ag/AgCl reference electrode and at scan rate 200 mV s^-1^ at room temperature. Supporting electrolyte is 0.1 M TBAPF_6_ in DMSO. The concentration of the compounds are 10^-3^ M.**

**Fig. (S11): Current *I_pa_* (**$\boldsymbol{\mu}$**A) versus the square root of the scan rate (mV/s) of PCHD ligand and its copper complex for the positive region (top) and the negative region (bottom) redox couples.**


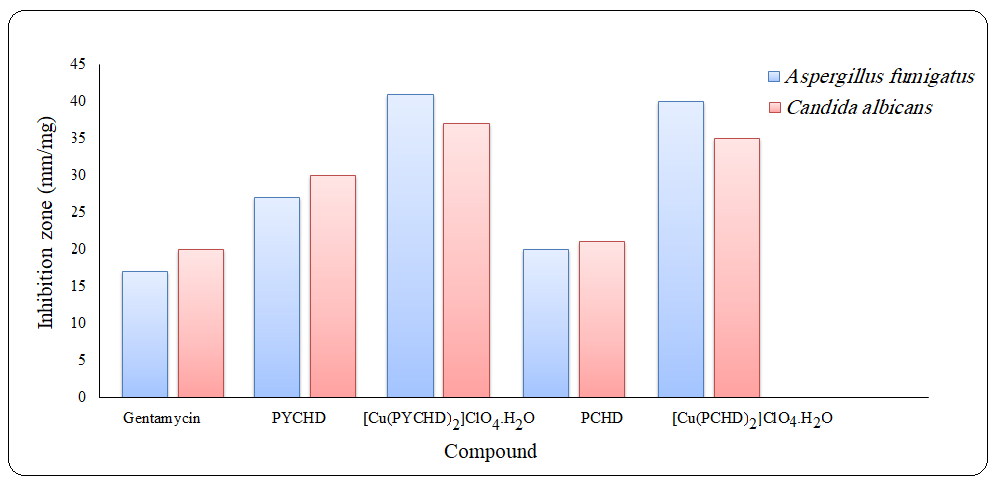


**Figure (S12): Antifungal activity representation of the synthesized ligands and their copper(I) complexes.**


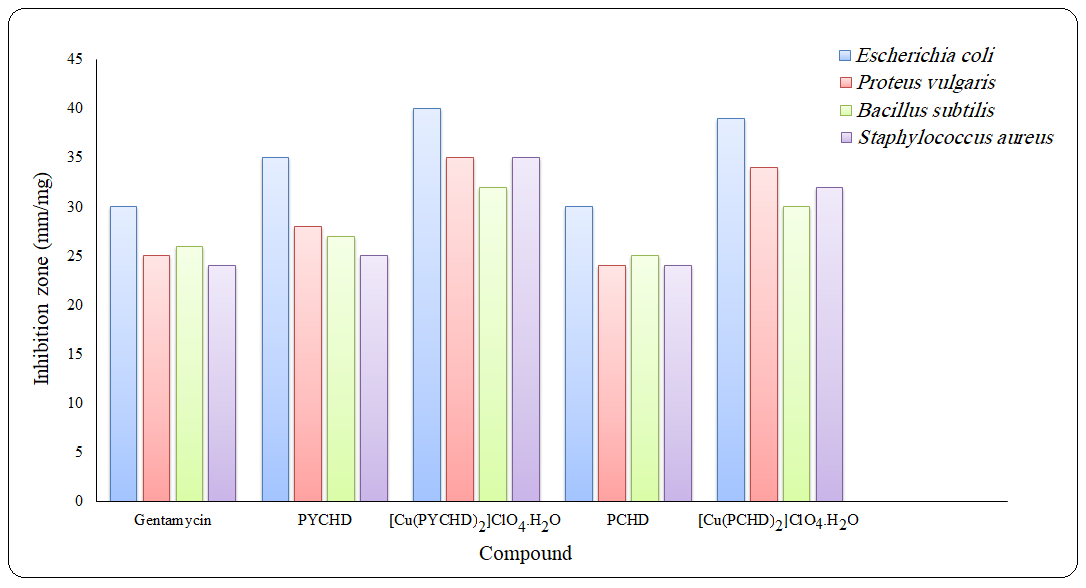


**Figure (S13): Antibacterial activity representation of the synthesized ligands and their copper(I) complexes.**


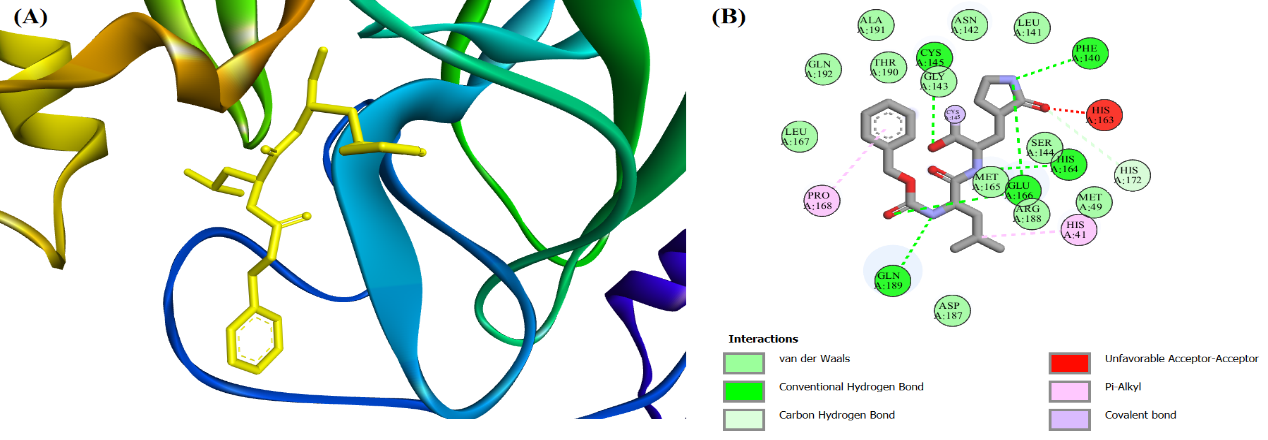


**Fig. (S14): Cartoon representation (A) and 2D mode of binding (B) of the SARS-CoV Mpro protein structures (6WTT) with K36 reference ligand inhibitor.**


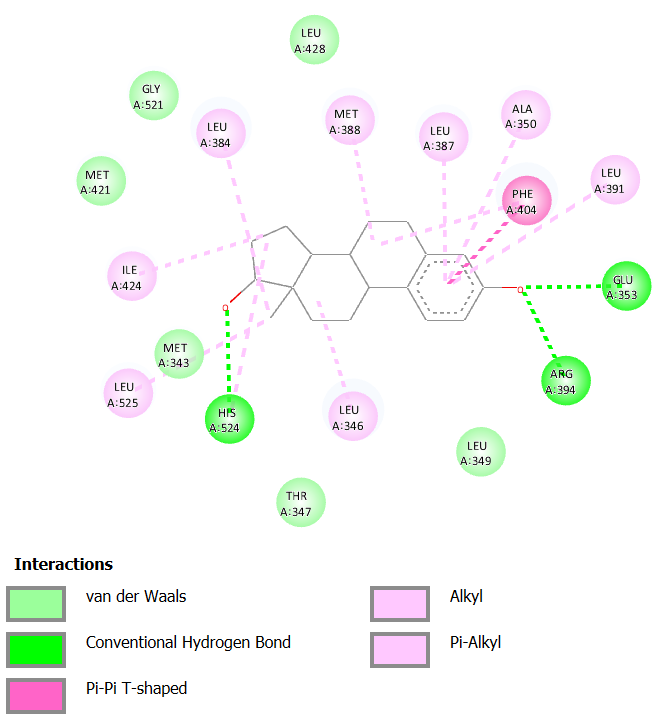


**Fig. (S15) : 2D mode of binding of Estrogen Receptor Alpha Ligand Binding Domain (6CBZ) with EST reference ligand inhibitor.**


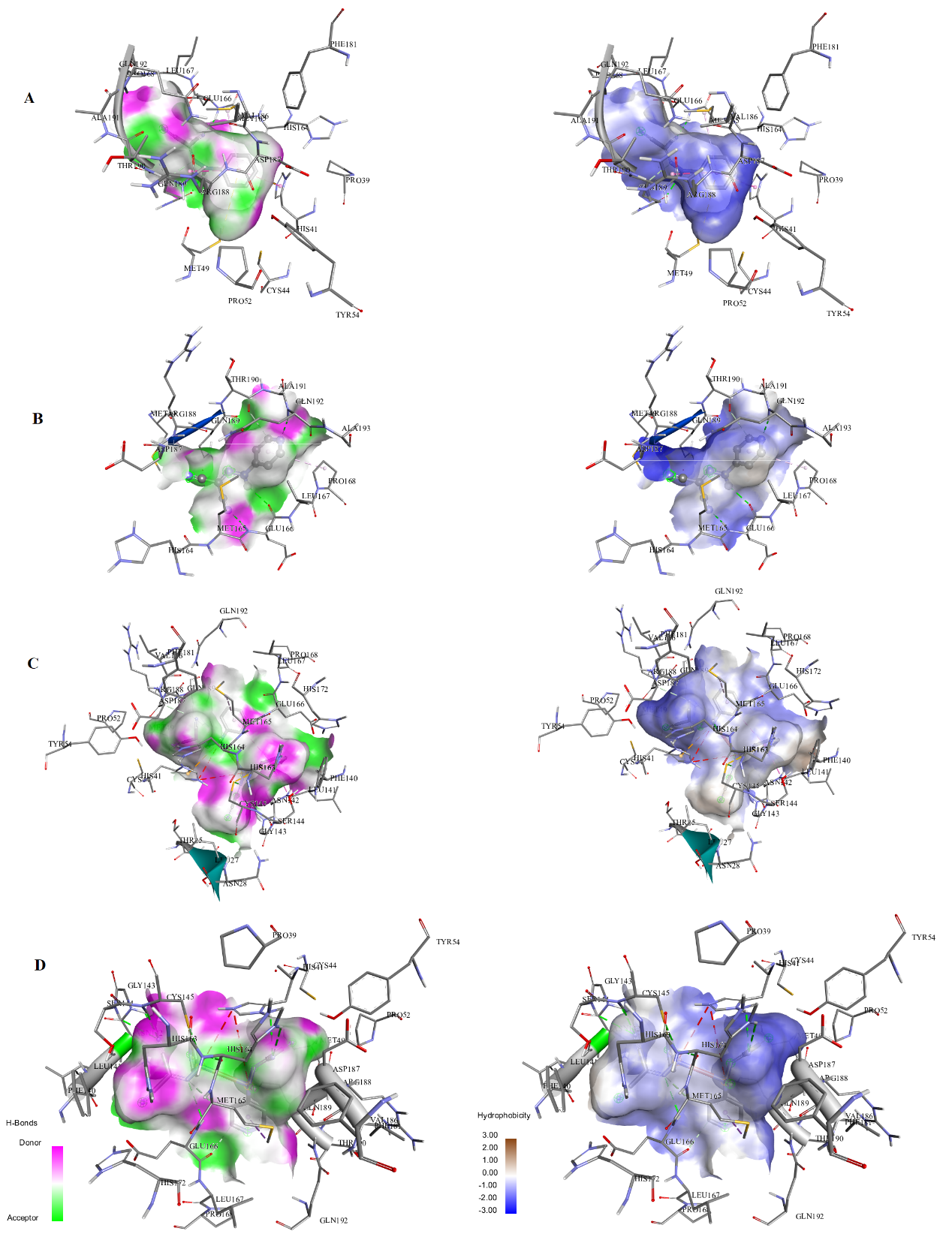


**Fig. (S16): H-bond and hydrophobicity interactions of the M^pro^ enzyme surface with (A) PCHD, (B) PyCHD, (C) [Cu(PCHD)_2_]ClO_4_.H_2_O, and (D)[Cu(PyCHD)_2_] ClO_4_.H_2_O (thick sticks represents the ligands and thin sticks are for amino acids)**

**Schemes**

**Scheme (S1):** **Suggested fragmentation pathways of phenylcarbonohydrazo-noyldicyanide ligand (PCHD)**

**Scheme (S2): Suggested fragmentation pathways of pyridin-4-ylcarbono hydrazonoyl dicyanide ligand (PyCHD)**.

**Scheme (S3): The suggested fragmentation pathways of [Cu(PCHD)_2_]ClO_4_. H_2_O.**

**Scheme (S4): The suggested fragmentation pathways of [Cu(PyCHD)_2_]ClO_4_.H_2_O.**

**Equations used for deducing the quantum chemical properties of synthesized compounds:**

χ = -(E_HOMO_ + E_LUMO_ )/2 (S2)

ɳ = (E_LUMO_ -E_HOMO_)/2 (S3)

σ = 1/ɳ , Pi = -χ (S4)

S = 1/2ɳ (S5)

ω = Pi^2^/2ɳ (S6)

Δ Nmax = (- Pi)/ɳ (S7)

### Tables

**Table (S1): The optimized molecular structure bond lengths of lignds and their copper complexes**

| **Bond length (A^o^)** | | | | | | | |
| --- | --- | --- | --- | --- | --- | --- | --- |
| **PCHD** | | **[Cu(PCHD)_2_]ClO_4_.H_2_O** | | **PyCHD** | | **[Cu(PyCHD)_2_]ClO_4_.H_2_O** | |
| C(1) - C(2) | 1.3913 | C(1) - C(2) | 1.4072 | C(1) - C(2) | 1.3908 | C(1) - C(2) | 1.4101 |
| C(1) - C(6) | 1.3935 | C(1) - C(6) | 1.4082 | C(1) - N(6) | 1.3376 | C(1) - H(6) | 1.0859 |
| C(1) - H(7) | 1.0836 | C(1) - H(7) | 1.0859 | C(1) - H(7) | 1.0858 | C(1)- N(37) | 1.3551 |
| C(2) - C(3) | 1.3975 | C(2) - C(3) | 1.4056 | C(2) - C(3) | 1.3951 | C(2) - C(3) | 1.4043 |
| C(2) - H(8) | 1.0853 | C(2) - H(8) | 1.0893 | C(2) - H(8) | 1.081 | C(2) - H(7) | 1.0878 |
| C(3) - C(4) | 1.3969 | C(3) - C(4) | 1.4039 | C(3) - C(4) | 1.396 | C(3) - C(4) | 1.4021 |
| C(3) - N(12) | 1.4119 | C(3) - N(12) | 1.4935 | C(3) - N(11) | 1.4048 | C(3)- N(10) | 1.4882 |
| C(4) - C(5) | 1.3907 | C(4) - C(5) | 1.4072 | C(4) - C(5) | 1.3913 | C(4) - C(5) | 1.4104 |
| C(4) - H(9) | 1.0815 | C(4) - H(9) | 1.0844 | C(4) - H(9) | 1.0846 | C(4) - H(8) | 1.0832 |
| C(5) - C(6) | 1.3954 | C(5) - C(6) | 1.409 | C(5) - N(6) | 1.3354 | C(5) - H(9) | 1.0859 |
| C(5) - H(10) | 1.0838 | C(5) - H(10) | 1.0861 | C(5) - H(10) | 1.0858 | C(5)- N(37) | 1.3563 |
| C(6) - H(11) | 1.0833 | C(6) - H(11) | 1.0858 | N(11)- N(13) | 1.3076 | N(10)-N(11) | 1.4356 |
| N(12) - N(14) | 1.3018 | N(12)- N(13) | 1.4301 | N(11)- H(12) | 1.0206 | N(10) - H(34) | 1.0341 |
| N(14) - C(15) | 1.3118 | N(13) - C(14) | 1.314 | N(13)- C(14) | 1.3074 | N(11) - C(12) | 1.3136 |
|  |  | N(12)-Cu(19) | 1.9951 |  |  | N(10)-Cu(17) | 1.9994 |
| C(15) - C(16) | 1.4288 |  |  | C(14) - C(15) | 1.4306 |  |  |
| N(12) - H(13) | 1.0209 | N(12) - H(38) | 1.0335 | C(14) - C(16) | 1.426 | C(12) - C(13) | 1.447 |
| C(15) - C(17) | 1.4247 | C(14) - C(15) | 1.4468 | C(15) - N(17) | 1.1568 | C(12) - C(14) | 1.4366 |
| C(16) - N(18) | 1.1574 | C(14) - C(16) | 1.4369 | C(16) - N(18) | 1.155 | C(13) - N(15) | 1.1819 |
| C(17) - N(19) | 1.1555 | C(15) - N(17) | 1.182 |  |  | C(14) - N(16) | 1.1806 |
|  |  | C(16) - N(18) | 1.1806 |  |  | Cu(17)-N(18) | 1.9995 |
|  |  | Cu(19)-N(20) | 1.9951 |  |  | N(18) - N(19) | 1.4356 |
|  |  | N(20) - N(21) | 1.4301 |  |  | N(18) - C(25) | 1.4882 |
|  |  | N(20) - C(27) | 1.4935 |  |  | N(18) - H(35) | 1.0341 |
|  |  | N(20) - H(39) | 1.0335 |  |  | N(19) - C(20) | 1.3136 |
|  |  | N(21) - C(22) | 1.314 |  |  | C(20) - C(21) | 1.447 |
|  |  | C(22) - C(23) | 1.4468 |  |  | C(20) - C(22) | 1.4366 |
|  |  | C(22) - C(24) | 1.4369 |  |  | C(21) - N(23) | 1.1819 |
|  |  | C(23) - N(25) | 1.182 |  |  | C(22) - N(24) | 1.1806 |
|  |  | C(24) - N(26) | 1.1806 |  |  | C(25) - C(26) | 1.4043 |
|  |  | C(27) - C(28) | 1.4056 |  |  | C(25) - C(27) | 1.4021 |
|  |  | C(27) - C(29) | 1.4039 |  |  | C(26) - C(28) | 1.4101 |
|  |  | C(28) - C(30) | 1.4072 |  |  | C(26) - H(29) | 1.0878 |
|  |  | C(28) - H(31) | 1.0893 |  |  | C(27) - C(30) | 1.4104 |
|  |  | C(29) - C(32) | 1.4072 |  |  | C(27) - H(31) | 1.0832 |
|  |  | C(29) - H(33) | 1.0844 |  |  | C(28) - H(32) | 1.0859 |
|  |  | C(30) - C(34) | 1.4082 |  |  | C(28) - N(36) | 1.3551 |
|  |  | C(30) - H(35) | 1.0859 |  |  | C(30) - H(33) | 1.0859 |
|  |  | C(32) - C(34) | 1.409 |  |  | C(30) - N(36) | 1.3563 |
|  |  | C(32) - H(36) | 1.0861 |  |  |  |  |
|  |  | C(34) - H(37) | 1.0858 |  |  |  |  |

**Table (S2): The optimized molecular structure bond angles of PCHD and its [Cu(PCHD)_2_]ClO_4_.H_2_O complex**

| **Bond Angle** | | | |
| --- | --- | --- | --- |
| **PCHD** | | **[Cu(PCHD)_2_]ClO_4_.H_2_O** | |
| C(2) - C(1) - H(7) | 119.4246 | C(2) - C(1) - H(7) | 119.5293 |
| C(6) - C(1) - C(7) | 120.278 | C(6) - C(1) - H(7) | 120.2912 |
| C(1) - C(2) - C(3) | 119.5657 | C(1) - C(2) - C(3) | 118.7287 |
| C(1) - C(2) - H(8) | 120.2333 | C(1) - C(2) - H(8) | 119.856 |
| C(3) - C(2) - H(8) | 120.2011 | C(3) - C(2) - H(8) | 121.408 |
| C(2) - C(3) - C(4) | 120.688 | C(2) - C(3) - C(4) | 122.1823 |
| C(2) - C(3) - N(12) | 117.7316 | C(2) - C(3) - N(12) | 117.1187 |
| C(4) - C(3) - N(12) | 121.5804 | C(4) - C(3) - N(12) | 120.6853 |
| C(3) - C(4) - C(5) | 118.9998 | C(3) - C(4) - C(5) | 118.2787 |
| C(3) - C(4) - H(9) | 119.8513 | C(3) - C(4) - H(9) | 120.8262 |
| C(5) - C(4) - H(9) | 121.1489 | C(5) - C(4) - H(9) | 120.8641 |
| C(4) - C(5) - C(6) | 120.8663 | C(4) - C(5) - C(6) | 120.6517 |
| C(4) - C(5) - H(10) | 119.1432 | C(4) - C(5) - H(10) | 119.3087 |
| C(6) - C(5) - H(10) | 119.9904 | C(6) - C(5) - H(10) | 120.0371 |
| C(1) - C(6) - C(5) | 119.5827 | C(1) - C(6) - C(5) | 119.9813 |
| C(1) - C(6) - H(11) | 120.1916 | C(1) - C(6) - H(11) | 119.9607 |
| C(5) - C(6) - H(11) | 120.2257 | C(5) - C(6) - H(11) | 120.0541 |
| C(3) - N(12) - H(13) | 117.6834 | C(3) - N(12) - N(13) | 113.4015 |
| C(3) - N(12) - N(14) | 122.1516 | C(3) - N(12) - Cu(19) | 102.1232 |
| H(13) - N(12) -N(14) | 120.165 | C(3) - N(12) - H(38) | 110.3442 |
| N(12) - N(14) - C(15) | 120.3192 | N(13) - N(12) - Cu(19) | 105.9841 |
| N(14) - C(15) - C(16) | 121.7969 | N(13) - N(12) - H(38) | 113.8865 |
| N(14) - C(15) - C(17) | 118.5387 | Cu(19) - N(12) - H(38) | 110.3339 |
| C(16) - C(15) - C(17) | 119.6644 | N(12) - N(13) - C(14) | 118.0583 |
|  |  | N(20) - Cu(19) - N(12) | 179.86316 |
|  |  | N(13) - C(14) - C(15) | 123.5027 |
|  |  | N(13) - C(14) - C(16) | 118.1697 |
|  |  | C(15) - C(14) - C(16) | 118.3209 |
|  |  | Cu(19) - N(20) - N(21) | 105.9878 |
|  |  | Cu(19) - N(20) - C(27) | 102.1156 |
|  |  | Cu(19) - N(20) - H(39) | 110.3366 |
|  |  | N(21) - N(20) - C(27) | 113.4006 |
|  |  | N(21) - N(20) - H(39) | 113.8876 |
|  |  | C(27) - N(20) - H(39) | 110.3446 |
|  |  | N(20) - N(21) - C(22) | 118.0591 |
|  |  | N(21) - C(22) - C(23) | 123.5011 |
|  |  | N(21) - C(22) - C(24) | 118.1694 |
|  |  | C(23) - C(22) - C(24) | 118.3227 |
|  |  | N(20) - C(27) - C(28) | 117.121 |
|  |  | N(20) - C(27) - C(29) | 120.6829 |
|  |  | C(28) - C(27) - C(29) | 122.1824 |
|  |  | C(27) - C(28) - C(30) | 118.7285 |
|  |  | C(27) - C(28) - H(31) | 121.4074 |
|  |  | C(30) - C(28) - H(31) | 119.8567 |
|  |  | C(27) - C(29) - C(32) | 118.2788 |
|  |  | C(27) - C(29) - H(33) | 120.8268 |
|  |  | C(32) - C(29) - H(33) | 120.8634 |
|  |  | C(28) - C(30) - C(34) | 120.176 |
|  |  | C(28) - C(30) - H(35) | 119.5298 |
|  |  | C(34) - C(30) - H(35) | 120.2906 |
|  |  | C(29) - C(32) - C(34) | 120.6513 |
|  |  | C(29) - C(32) - H(36) | 119.3085 |
|  |  | C(34) - C(32) - H(36) | 120.0376 |
|  |  | C(30) - C(34) - C(32) | 119.9815 |
|  |  | C(30) - C(34) - H(37) | 119.9601 |
|  |  | C(32) - C(34) - H(37) | 120.0545 |

**Table (S3): The optimized molecular structure bond angles of PyCHD and its [Cu(PyCHD)_2_]ClO_4_.H_2_O complex**

| **Bond Angle** | | | |
| --- | --- | --- | --- |
| **PyCHD** | | **[Cu(PyCHD)_2_]ClO_4_.H_2_O** | |
| C(2) - C(1) - N(6) | 124.4332 | C(2) - C(1) - H(6) | 120.5109 |
| C(2) - C(1) - H(7) | 119.4889 | C(2) - C(1) - N(37) | 123.143 |
| N(6) - C(1) - H(7) | 116.0779 | H(6) - C(1) - N(37) | 116.3449 |
| C(1) - C(2) - C(3) | 117.5913 | C(1) - C(2) - C(3) | 117.4299 |
| C(1) - C(2) - H(8) | 121.3013 | C(1) - C(2) - H(7) | 119.8499 |
| C(3) - C(2) - H(8) | 121.1074 | C(3) - C(2) - H(7) | 122.7145 |
| C(2) - C(3) - C(4) | 119.0677 | C(2) - C(3) - C(4) | 120.7817 |
| C(2) - C(3) - N(11) | 122.3116 | C(2) - C(3) - N(10) | 117.8245 |
| C(4) - C(3) - N(11) | 118.6208 | C(4) - C(3) - N(10) | 121.3782 |
| C(3) - C(4) - C(5) | 118.0768 | C(3) - C(4) - C(5) | 117.0968 |
| C(3) - C(4) - H(9) | 121.4638 | C(3) - C(4) - H(8) | 122.0422 |
| C(5) - C(4) - H(9) | 120.4594 | C(5) - C(4) - H(8) | 120.837 |
| C(4) - C(5) - C(6) | 123.9312 | C(4) - C(5) - H(9) | 120.3533 |
| C(4) - C(5) - H(10) | 119.7247 | C(4) - C(5) - N(37) | 123.5009 |
| N(6) - C(5) - H(10) | 116.3441 | H(9) - C(5) - N(37) | 116.1449 |
| C(1) - N(6) - C(5) | 116.8999 | C(3) - N(10) - N(11) | 112.8375 |
| C(3) - N(11) - H(12) | 118.0189 | C(3) - N(10) - Cu(17) | 103.2312 |
| C(3) - N(11) - N(13) | 121.6445 | C(3) - N(10) - H(34) | 110.4024 |
| H(12) - N(11) - N(13) | 120.3366 | N(11) - N(10) - Cu(17) | 105.952 |
| N(11) - N(13) - C(14) | 120.1729 | N(11) - N(10) - H(34) | 113.8454 |
| N(13) - C(14) - C(15) | 121.953 | Cu(17) - N(10) - H(34) | 109.9078 |
| N(13) - C(14) - C(16) | 118.5609 | N(10) - N(11) - C(12) | 117.8248 |
| C(15) - C(14) - C(16) | 119.4861 | N(11) - C(12) - C(13) | 123.4292 |
|  |  | N(11) - C(12) - N(14) | 118.1891 |
|  |  | C(13) - C(12) - C(14) | 118.3733 |
|  |  | N(10) - Cu(17) - N(18) | 179.67629 |
|  |  | N(18) - Cu(17) - N(11) | 109.9078 |
|  |  | Cu(17) - N(18) - N(19) | 105.9506 |
|  |  | Cu(17) - N(18) - C(25) | 103.2318 |
|  |  | Cu(17) - N(18) - H(35) | 109.9077 |
|  |  | N(19) - N(18) - C(25) | 112.8382 |
|  |  | N(19) - N(18) - H(35) | 113.8451 |
|  |  | C(25) - N(18) - H(35) | 110.4029 |
|  |  | N(18) - N(19) - C(20) | 117.8246 |
|  |  | N(19) - C(20) - H(21) | 123.4292 |
|  |  | N(19) - C(20) - C(22) | 118.1892 |
|  |  | H(21) - C(20) - C(22) | 118.3734 |
|  |  | N(18) - C(25) - C(26) | 117.8241 |
|  |  | N(18) - C(25) - C(27) | 121.3788 |
|  |  | C(26) - C(25) - C(27) | 120.7815 |
|  |  | C(25) - C(26) - C(28) | 117.4301 |
|  |  | C(25) - C(26) - H(29) | 122.7143 |
|  |  | C(28) - C(26) - H(29) | 119.85 |
|  |  | C(25) - C(27) - C(30) | 117.0968 |
|  |  | C(25) - C(27) - H(31) | 122.0422 |
|  |  | C(30) - C(27) - H(31) | 120.837 |
|  |  | C(26) - C(28) - H(32) | 120.511 |
|  |  | C(26) - C(28) - N(36) | 123.1429 |
|  |  | H(32) - C(28) - N(36) | 116.3448 |
|  |  | C(27) - C(30) - H(33) | 120.3532 |
|  |  | C(27) - C(30) - N(36) | 123.501 |
|  |  | H(33) - C(30) - N(36) | 116.1449 |
|  |  | C(28) - N(36) - C(30) | 118.0429 |
|  |  | C(1) - C(37) - C(5) | 118.043 |

**Table (S4) : The minimum inhibitory concentration (MIC, μg/ml) of synthesized ligands, their copper complexes and the reference drugs.**

| Compound | **Gram Negative** | | **Gram Positive** | | **Fungi** | |
| --- | --- | --- | --- | --- | --- | --- |
|  | ***E.Coli*** | ***P.Vulgaris*** | ***B.Subtilis*** | ***S.Aureus*** | ***A. Fumigatus*** | ***C.Albicans*** |
| PCHD | 4.21 | 6.91 | 4.75 | 11.01 | 166.89 | 320.00 |
| [Cu(PCHD)_2_]ClO_4_.H_2_O | 3.15 | 3.56 | 3.83 | 8.92 | 151.73 | 307.00 |
| PyCHD | 3.99 | 4.62 | 4.02 | 9.11 | 153.22 | 310.13 |
| [Cu(PyCHD)_2_]ClO_4_.H_2_O | 2.11 | 2.40 | 3.80 | 7.64 | 151.08 | 305.35 |
| Gentamycin | 4.80 | 4.80 | 4.80 | 9.70 | -- | -- |
| Ketoconazole | -- | -- | -- | -- | 156.25 | 312.50 |

**Table (S5): Summary of the compounds interacting residues of corona virus Mpro protein and** **Estrogen Receptor Alpha Ligand Binding Domain.**

| **Receptor**  **(PDB id)** | **Compounds** | **Hydrogen and electrostatic bonds** | | | | **Hydrophobic interactions** | **van der Waals interactions** |
| --- | --- | --- | --- | --- | --- | --- | --- |
|  |  | **Mpro** | | **Atom in bio-active compound** | **Distance (Å)** |  |  |
|  |  | **Amino acid** | **Atom** |  |  |  |  |
| **6CBZ** | **PCHD** | ARG394  LEU346 | H  O | N  H | 2.15  2.03 | ALA350  LEU384  LEU525 | TRP 383, MET 343  LEU 387, THR 347  GLU 353, LEU 349  LEU 391, PHE 404 |
|  | **[Cu(PCHD)_2_]ClO_4_.H_2_O** | LEU346  GLU353 | O  O | H  N | 2.00  5.39 | LEU349  ALA350  LEU387  MET421  MET343 | LEU525, HIS 524  LEU 540,TRP 383  THR 347, LEU 384  LEU 428, LEU 391  MET 388, PHE 425  PHE 404,ARG 394  ILE 424, GLY 521 |
|  | **PyCHD** | ARG394  LEU346 | H  O | N  H | 2.17  2.06 | ALA350  LEU384  LEU525 | THR 347, LEU 387  GLU 353, LEU 391  PHE 404, LEU 349  LEU 540, TRP 383 |
|  | **[Cu(PyCHD)_2_]ClO_4_.H_2_O** | ARG394  LEU346  GLU353 | H  O  O | N  H  N | 2.65  2.02  5.38 | LEU349  ALA350  LEU387  MET421 | LEU 384, HIS 524  GLY 521, PHE 425  ILE 424, MET 388  LEU 428, MET 343  THR 347, LEU 391  TRP 383, LEU 540  PHE 404, LEU 525 |
|  | | | | | | | |
| **6 WTT** | **PCHD** | GLU166  GLN189  GLN189 | HN  O  C | N  H  N | 2.15  2.01  3.02 | HIS41  MET49  HIS41  ARG188  GLN189  MET165 | GLN 192  THR 190  HIS 164  ASP 187  TYR 54 |
|  | **[Cu(PCHD)_2_]ClO_4_.H_2_O** | CYS145  HIS164  ASP187 | S  O  C | N  H  N | 2.97  2.16  3.19 | HIS41  GLU166  CYS145  LEU141  ASN142  MET165 | PHE 140, HIS 163  SER 144, LEU 27  GLY 143,THR 26  CYS 44, MET 49  TYR 54, ARG 188  GLN 189, PHE 181 |
|  | **PyCHD** | GLU166  GLN192  GLU166  THR190 | HN  HN  O  O | N  N  H  C | 2.11  2.15  2.47  3.25 | PRO168 | ARG 188, LEU 167  GLN 189, MET 165  ALA 191 |
|  | **[Cu(PyCHD)_2_]ClO_4_.H_2_O** | HIS41  GLY143  SER144  CYS145  HIS164  GLU166  CYS145  HIS164  MET165 GLU166 | H  HN  HN  HN  H  HN  S  O  C  O | N  N  N  N  N  H  H  H  N  C | 2.63  2.74  2.94  2.80  2.99  3.09  2.69  2.79  3.28  3.07 | MET165  CYS145 | GLN 189, GLN 192  ARG 188, PHE 181  MET 49, ASP 187  TYR 54, CYS 44  PRO 52, LEU 141  HIS 163, PHE 140  HIS 172, ASN 142 |

**References**

[1] J. Bassett, R. Denney, G. Jeffery, J. Mendham, Textbook of quantitative inorganic analysis including elementary instrumental analysis, (1978).

[2] H.R. Jousimies-Somer, S.M. Finegold, Problems encountered in clinical anaerobic bacteriology, Reviews of infectious diseases 6(Supplement_1) (1984) S45-S50.

[3] A.t. Bauer, Antibiotic susceptibility testing by a standardized single disc method, Am J clin pathol 45 (1966) 149-158.

[4] V. Sumalatha, S. Daravath, A. Rambabu, G. Ramesh, Antioxidant, antimicrobial, DNA binding and cleavage studies of novel Co (II), Ni (II) and Cu (II) complexes of N, O donor Schiff bases: Synthesis and spectral characterization, Journal of Molecular Structure 1229 (2021) 129606.

1. 1 Corresponding Author, E-mail; [eman.hassan_pg@alexu.edu.eg](mailto:eman.hassan_pg@alexu.edu.eg); [biochemisteman_hassan@yahoo.com](mailto:biochemisteman_hassan@yahoo.com) [↑](#footnote-ref-1)
